# Functional Characterization of Multidomain LPMOs from Marine *Vibrio* Species Reveals Modulation of Enzyme Activity by Domain–Domain Interactions

**DOI:** 10.1021/acs.biochem.5c00529

**Published:** 2025-12-12

**Authors:** Yong Zhou, Eirik G. Kommedal, Zarah Forsberg, Gustav Vaaje-Kolstad, Wipa Suginta, Vincent G. H. Eijsink

**Affiliations:** † School of Biomolecular Science and Engineering (BSE), 423058 Vidyasirimedhi Institute of Science and Technology (VISTEC), Rayong 21210, Thailand; ‡ Faculty of Chemistry, Biotechnology and Food Science (KBM), 56625 Norwegian University of Life Sciences (NMBU), Ås 1432, Norway; § Agricultural Genomics Institute at Shenzhen (AGIS), Chinese Academy of Agricultural Sciences (CAAS), Shenzhen 518120, China

## Abstract

Several bacterial pathogens secrete multidomain enzymes known as lytic polysaccharide monooxygenases (LPMOs) that are important for virulence. One example is the *Vibrio cholerae* virulence factor GbpA (*Vc*GbpA), in which an N-terminal LPMO domain is followed by two domains of unknown function called GbpA2 and GbpA3, and a C-terminal chitin-binding domain called CBM73. In-depth functional characterization of full-length and truncated variants of *Vc*GbpA and a homologue from *V. campbellii* (previously *V. harveyi*, *Vh*GbpA) showed that the catalytic LPMO domains of these proteins exhibit properties similar to natural single-domain LPMOs with established roles in chitin degradation. Interestingly, binding to chitin and efficient degradation of this substrate were affected by the presence of the GbpA2 and GbpA3 domains. Combined with structural predictions and analyses of sequence conservation, our data show that GbpA3 has evolved to interact with the reduced catalytic copper site in the LPMO domain to prevent off-pathway reactions in the absence of substrate. Substrate binding by CBM73 weakens this interaction, enabling the activation of the LPMO only when substrate is present. These observations shed new light into the functionality of these multidomain LPMOs and uncover a novel mechanism for regulating LPMO activity.

## Graphical abstract

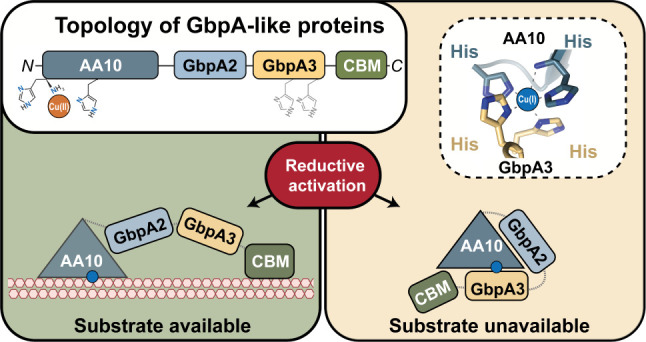

## Introduction

Chitin is a natural amino polysaccharide composed of β-(1,4)-linked *N*-acetyl-d-glucosamine (GlcNAc).^1–3^ Single chitin chains pack together to form insoluble crystalline polysaccharide microfibrils that confer mechanical stability to fungal cell walls and the exoskeletons of crustaceans and insects.^2^ Chitin may occur in complex copolymeric structures, such as fungal cell walls, which also contain glucans, mannans, and glycoproteins.^4,5^ Enzymatic conversion of chitin is of interest for many reasons: chitin is an abundant bioresource that can be valorized;^6^ chitin turnover is important for the global carbon cycle, especially in aquatic ecosystems;^7^ and chitin-degrading enzyme systems are associated with a wide variety of host–pathogen interactions and with microbial competition.^8,9,77^ Some microorganisms have evolved complex enzymatic systems to depolymerize chitin to GlcNAc, which can be utilized as a nutrient source. Next to endo- and exoacting glycoside hydrolases (chitinases), these systems include lytic polysaccharide monooxygenases (LPMOs), which catalyze oxidative cleavage of glycosidic bonds in crystalline chitin microfibrils, thus providing new access points for further depolymerization by hydrolytic enzymes.^13–17^

LPMOs (EC 1.14.99.53–56) are monocopper enzymes that utilize hydrogen peroxide (H_2_O_2_) to oxidize the glycosidic bonds of chitin and other crystalline polysaccharides.^14,18–21^ Since the discovery of their catalytic activity in 2010,^14^ LPMOs have received considerable attention due to their intriguing catalytic mechanism^20–25^ and their importance for industrial biorefineries.^26–28^ The CAZy database currently contains eight sequence-based LPMO families, which are referred to as Auxiliary Activities (AA), with family AA10 containing bacterial chitin-active LPMOs. The single catalytic copper ion of LPMOs is coordinated by a conserved histidine brace^18^ and copper reactivity is modulated by nearby amino acids in what is called the second coordination sphere.^29,30^ LPMOs were originally considered monooxygenases (R–H + O_2_ + 2e^–^ + 2H^+^ → R–OH + 2H_2_O) employing O_2_ as cosubstrate.^14,18,19^ Recent works have demonstrated that LPMOs operate as peroxygenases (R–H + H_2_O_2_ → R–OH + H_2_O) using H_2_O_2_ as cosubstrate.^20,21,31–35^ In both reactions, the LPMO needs to be reduced by a reductant; in the monooxygenase reaction, further supply of electrons and protons is required during catalysis, whereas these are delivered in the form of H_2_O_2_ in the peroxygenase reaction. Importantly, for convenience, LPMO reactions are frequently run under apparent monooxygenase conditions (LPMO + substrate + reductant, aerobic), i.e., without exogenously added H_2_O_2_. In such “reductant-driven” reactions, LPMO activity is limited by the rate of *in situ* H_2_O_2_ generation resulting from LPMO-catalyzed or abiotic oxidation of the reductant.^36–39^ These reductant-driven reactions are usually slow, while reactions supplied with H_2_O_2_ are orders of magnitude faster.^31–35^

Importantly, high concentrations of H_2_O_2_, which may emerge as a result of both *in situ* production or too high levels of H_2_O_2_ feeding, may lead to autocatalytic enzyme inactivation, especially at a low substrate concentration.^20,40^ A considerable fraction of LPMOs contain a carbohydrate-binding module (CBM) that promotes substrate binding. By doing so, CBMs increase the chance that available H_2_O_2_ is used to productively cleave the substrate through a peroxygenase reaction, rather than nonproductively in a futile peroxidase reaction that may lead to enzyme damage.^20,41,42^ It has been pointed out that, in reactions driven by ascorbate oxidation, enzyme inactivation may be a self-reinforcing process:^43^ as the LPMO becomes damaged, copper is released,^44^ promoting abiotic ascorbate oxidation and further increasing H_2_O_2_ levels, thereby accelerating enzyme inactivation.

In recent years, several multimodular LPMOs have been described.^12,45–51^ Some of these multimodular LPMOs contain both a CBM and a glycoside hydrolase domain with similar substrate specificity as the LPMO domain, and their role in biomass conversion thus seems obvious.^46,47^ On the other hand, intriguingly, there is a large group of bacterial LPMOs that comprise one or two domains of unknown function in between an N-terminal chitin-active LPMO domain and a C-terminal chitin-binding CBM, most often a CBM5/12 or CBM73. Both expression studies and the ecological niches in which these enzymes are found do not necessarily point to a role of these LPMOs in chitin conversion,^48^ and one may wonder if chitin is the biologically relevant substrate. Moreover, these LPMOs are often found in bacterial pathogens, and for several of these, a clear connection with virulence has been established.^10,12^

One example of these enzymes is the four-domain GbpA from *Vibrio cholerae* (*Vc*GbpA), which is a known virulence factor that was originally referred to as a GlcNAc-binding protein A, hence the abbreviation GbpA.^11,45^ *Vc*GbpA consists of a chitin-active LPMO domain,^52^ two internal domains referred to as GbpA2 and GbpA3, and a C-terminal CBM73 domain with known affinity for chitin.^53^ Structurally, the chitin-active LPMO domain is similar to other known chitin-active LPMOs such as *Sm*AA10A (originally known as CBP21), the chitin-active LPMO from *S. marcescens*.^14^ Recently, the complete structure of a homologous four-domain protein from *V. campbellii* (previously known as *V. harveyi*, *Vh*GbpA) was determined by X-ray crystallography (Figure 1A), showing a rather elongated overall conformation, compatible with SAXS data for both *Vc*GbpA and *Vh*GbpA.^45,50^ While the functional roles of the GbpA2 and GbpA3 remain unknown, it has been suggested that they may be involved in the interaction between *Vibrio* and relevant host surfaces.^13,54^

**Figure 1:**
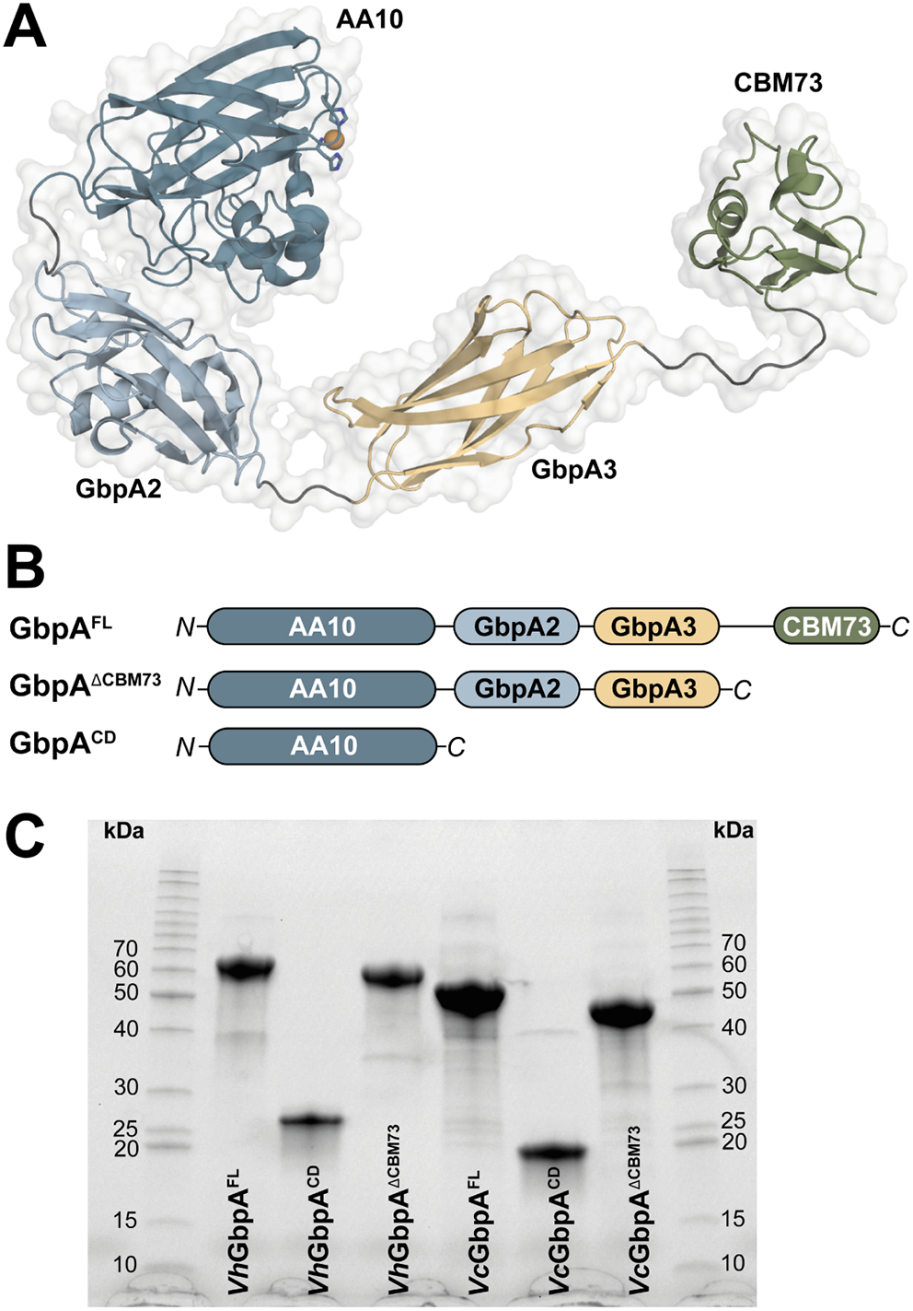
Overview of GbpA variants. (A) Structure of full-length *Vibrio campbellii* GbpA (*Vh*GbpA^FL^) as determined by X-ray crystallography (PDB ID: 8GUL), illustrating the modular architecture of the protein; the catalytic copper appears as an orange sphere. (B) Schematic representation of the domain organization of full-length GbpA proteins and the two truncated variants generated in this study for both *V. cholerae* (*Vc*GbpA) and *V. campbellii* (*Vh*GbpA). (C) SDS-PAGE analysis of purified protein variants, i.e., full-length and truncated variants for *Vh*GbpA (left) and *Vc*GbpA (right).

To gain additional insight into the catalytic abilities of GbpA-like proteins and to investigate the roles of their individual domains, we have carried out an in-depth functional characterization of full-length *Vh*GbpA, as well as two truncated variants: *Vh*GbpA^CD^, which contains only the catalytic LPMO domain, and *Vh*GbpA^ΔCBM73^, which lacks the CBM73 domain. We studied multiple enzyme properties, such as binding to chitin, reduction and reoxidation rates, and the efficiency of on-and-off pathway reactions. Several of these experiments were also done with similar variants of *Vc*GbpA, showing similar results and adding confidence to the conclusions. Unexpectedly, the comparison of wild-type enzymes with truncated variants revealed an interaction between the combined GbpA2 and GbpA3 part of the protein and the catalytic domain. Structure predictions with AlphaFold3 ^55^ and sequence conservation patterns strongly suggest a copper-mediated interaction between GbpA3 and the catalytic copper site, which seems to have evolved to protect the enzyme from engaging in damaging off-pathway reactions. These observations lend support to a novel model in which *Vibrio* species regulate the activity of this virulence factor in a substrate-dependent manner.

## Results and Discussion

### Protein Production and Assessment of Redox Properties

The two wild-type GbpA proteins and two similarly truncated variants of each were produced and purified (Figure 1), yielding 4.5–25 mg of purified copper-saturated protein per liter of culture. Of note, in the below, the *Vc*GbpA and *Vh*GbpA variants generally show similar features despite the presence of a His-tag on the *Vh*GbpA variants, adding confidence to the results. For the *Vh*GbpA variants, protein integrity was assessed by determining apparent melting temperatures. All three proteins showed a clear unfolding transition with apparent melting temperatures between 45 and 50 °C in reactions with EDTA and between 50 and 60 °C in reactions without EDTA (Figure S1). Copper binding is known to stabilize correctly folded LPMOs; hence, the observed destabilizing effect of EDTA was expected and indicates that the proteins do bind copper. Accordingly, all protein variants showed reductant-dependent activity on β-chitin as demonstrated by MALDI-ToF MS analysis of solubilized reaction products (Figures S2 and S3).

The possible effects of the truncations on the reactivity of the copper site were assessed using a variety of methods. Measurements of the oxidase activity i.e., ascorbate-dependent production of H_2_O_2_ in the absence of substrate,^43,56^ gave rates varying from 0.15 to 0.66 min^–1^ (Table S1), which are similar to the rates observed for other chitin-active bacterial LPMOs.^43,57^ Interestingly, for both *Vh*GbpA and *Vc*GbpA, the full-length enzyme and the CBM73-truncated enzyme, i.e., the two enzyme variants containing the GbpA2 and GbpA3 domains, showed 2–3 times lower oxidase rates than the isolated catalytic domain (see below for further discussion).

The second-order rates for reduction with ascorbic acid and reoxidation with H_2_O_2_ in the absence of substrate were determined using stopped-flow fluorimetry. This approach was based on observations by Bissaro et al.,^58^ who showed that the fluorescence signal of an LPMO depends on the redox state of the copper center (Figure S4). Experiments were done using pseudo-first-order conditions using different concentrations of AscA in a single-mixing experiment to assess reduction, or H_2_O_2_, in a double-mixing experiment, to assess reoxidation (see Materials and Methods section for details). For each protein variant, the total fluorescence change upon reduction was the same for all tested AscA concentrations, suggesting that reduction was complete in all cases. The reduction reaction remained monophasic but increased in rate with increasing concentrations of AscA. Likewise, the reoxidation reaction remained monophasic but increased in rate with rising H_2_O_2_ concentrations. Each fluorescence-versus-time curve was fitted to a single exponential function to derive pseudo-first-order rate constants (*k*_obs_), which were plotted as a function of [AscA] or [H_2_O_2_]. For both assays, the data could be fitted to a straight line, yielding the second-order rate constants *k*_AscA_ and *k*_H_2_O_2__ (Figure S4). The results (Table 1) show similar reduction (1.14–1.96 × 10^5^ M^–1^·s^–1^) and reoxidation (5.71–9.28 × 10^3^ M^–1^·s^–1^) rates for all protein variants. Although rate differences were small, it may be noted that the variants only comprising the catalytic domain showed the lowest rate of reduction and the highest rate of reoxidation for both *Vh*GbpA and *Vc*GbpA.

**Table 1:** Reduction and Reoxidation Rates^a^

	Reduction rate (*k*_AscA_, 10^3^ M^–1^·s^**–**1^)			Reoxidation rate (*k*_H_2_O_2__, 10^3^ M^–1^·s^**–**1^)		
	Full-length	AA10 CD	ΔCBM73	Full-length	AA10 CD	ΔCBM73
*Vh*GbpA	155 ± 2	114 ± 2	182 ± 2	5.7 ± 0.1	8.9 ± 0.0	8.4 ± 0.1
*Vc*GbpA	196 ± 7	128 ± 2	138 ± 1	8.5 ± 0.2	9.3 ± 0.1	7.0 ± 0.1

a The measurements were performed in 20 mm Tris-HCl, pH 7.5. The reported values are derived from three independent experiments and are presented with standard deviations. Note that the method used for measuring reoxidation of *Vh*GbpA^FL^ eviates from the method used in all other cases; see Materials and Methods section and Figure S4, which shows the underlying kinetic curves.

The kinetic values derived from the stopped-flow experiments provide unprecedented insight into the redox properties of GbpA-type LPMOs. Our data show that the redox properties of *Vc*GbpA and *Vh*GbpA are very similar. Moreover, the reduction and reoxidation rates obtained for these to GbpA’s are similar to those obtained for the well-characterized bacterial chitin-active LPMO *Sm*AA10A (or CBP21) at pH 7.0.^59^ Considering the obtained second-order rate constants for reduction by AscA, even the lowest AscA concentration employed in this study (25 μM) would yield a rate of reduction of 3–4 s^–1^, which is similar to the *k*_cat_ of 6.7 s^–1^ determined for the peroxygenase reaction catalyzed by *Sm*AA10A^33^ and orders of magnitude higher than the rate of the oxidase reaction. Thus, it is highly unlikely that reduction is rate-limiting in any of the experiments described here.

For comparison, we also assessed reoxidation by O_2_. As expected, based on the observed rates of the oxidase reaction (0.15–0.66 min^–1^; see above) and earlier studies (e.g. ref 59), reoxidation by O_2_ was found to be exceedingly slow at ambient conditions (approximately 250 μM O_2_) (Figure S5). Full reoxidation required up to 1 h in these conditions (Figure S5) in contrast to reoxidation by H_2_O_2_ which occurs in less than 20 s at the lowest H_2_O_2_ concentration (25 μM H_2_O_2_). Of note, rate constants for reoxidation for binuclear copper enzymes such as dopamine monooxygenase^60^ and galactose oxidase,^61^ as well as for certain multicopper laccases^62^ by O_2_ amount to 10^5^ to 10^7^ M^–1^·s^–1^, which is faster than the rate constants determined for reoxidation of the LPMO by H_2_O_2_ (Table 1).

All in all, these data show that *Vc*GbpA and *Vh*GbpA are very similar enzymes and that the copper reactivity of their LPMO domain is similar to that of other chitin-active bacterial LPMOs. The data further suggest that truncation of the CBM hardly affects copper reactivity, whereas truncation of all three additional domains does seem to modulate this reactivity to some extent. This is discussed further below.

### Binding to Chitin

Previous studies have shown that *Vc*GbpA binds *N*-acetylglucosamine (GlcNAc)-containing carbohydrates^11^ including different forms of chitin.^45^ To investigate whether this extends to *Vh*GbpA and to assess the possible impact of the different domains, we studied the binding of the various *Vc*GbpA and *Vh*GbpA variants to chitin in the absence and presence of ascorbate. From earlier studies, it is known that reduction of the LPMO promotes substrate binding.^63,64^ Binding to α-chitin was weak in all cases, even in the presence of ascorbate, preventing reliable comparison of the variants (results not shown). In contrast, the binding studies with β-chitin revealed clear binding and, moreover, revealed some remarkable differences between the enzyme variants (Figure 2). As expected, the presence of reductant promoted the binding of *Vh*GbpA^FL^ and *Vh*GbpA^CD^, but this effect was absent for *Vh*GbpA^ΔCBM73^ (Figure 2A,B). The *Vh*GbpA^ΔCBM73^ variant bound weakly to the substrate, regardless of the presence of a reductant. Analysis of binding to β-chitin was also analyzed over time for variants of both *Vh*GbpA and *Vc*GbpA. The binding curves (Figure 2C,D) show rapid binding within 1 min, which was the shortest possible sampling interval, and confirmed the remarkable observation described above: for both *Vh*GbpA and *Vc*GbpA, the CBM73-truncated variant showed weaker binding to chitin, than the catalytic domain only (Figure 2C,D).

**Figure 2:**
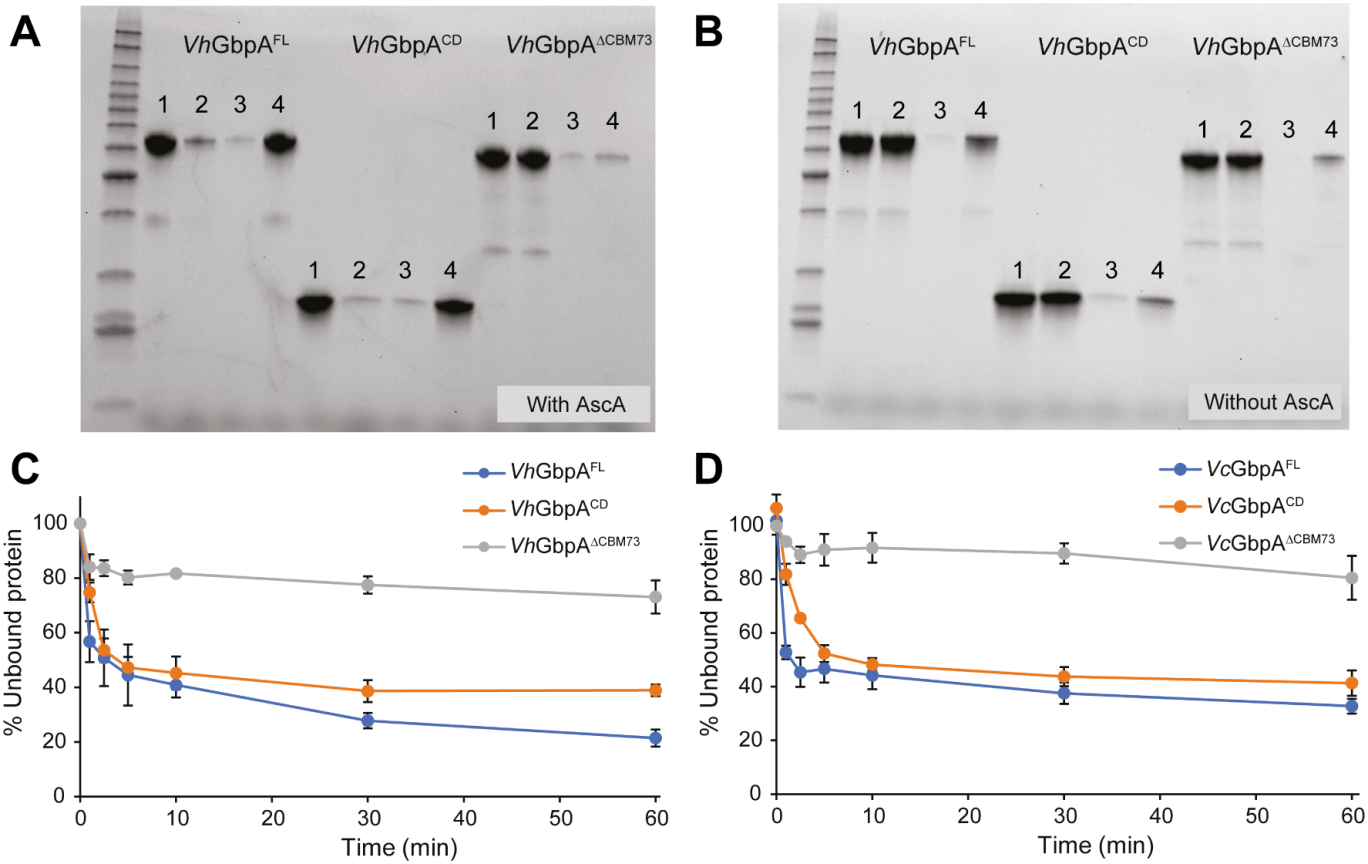
Binding of *Vh*GbpA and *Vc*GbpA variants to β-chitin. (A, B) Cu­(II)-loaded *Vh*GbpA variants (10 μM for *Vh*GbpA and *Vh*GbpA^ΔCBM73^, 20 μM for *Vh*GbpA^CD^) were incubated with 10 g·L^–1^ β-chitin in the presence (A) or absence (B) of 1 mM ascorbic acid in 20 mM Tris-HCl, pH 7.5, at 22 °C, 1000 rpm for 1 h. Subsequently, the chitin-bound enzyme was separated from the unbound fraction by centrifugation. The chitin fraction was washed twice with buffer and then resolubilized in an equal volume of SDS-PAGE sample buffer. The protein content in each fraction was qualitatively analyzed by SDS-PAGE. For each binding reaction, four samples are shown: 1, supernatant of a reaction without chitin; 2, supernatant of a reaction with chitin; 3, supernatant from the second washing step; 4, protein in the washed chitin pellet (this protein was solubilized by incubating the pellet in SDS-PAGE sample buffer at 95 °C). (C, D) Binding of *Vh*GbpA and *Vc*GbpA variants to β-chitin over time in the presence of ascorbic acid. The reactions were performed in 20 mM Tris-HCl, pH 7.5, at 22 °C, 1000 rpm using 2 μM enzyme, 10 g·L^–1^ β-chitin. In this experiment, the nonbound enzyme was separated from the insoluble chitin fraction by filtration using a vacuum manifold and quantified in 96-well microtiter plates using a modified Bradford assay.^67^

These results are unexpected for several reasons. First, the fact that the full-length enzyme and the catalytic domain only bind approximately equally strongly to chitin is unexpected, considering that the latter lacks the CBM73 with affinity for chitin^53^ and considering previous studies showing that chitin-binding CBMs enhance substrate binding by chitin-active LPMOs.^41^ On the other hand, some, but not all, single LPMO domains are known to bind well to their substrate, such as *Sm*AA10A.^65,66^ Second, and most remarkably, the removal of the CBM73 somehow prevents the binding of the reduced catalytic domain to chitin. This may be taken to suggest that in the CBM73-truncated variant, the two internal domains, GbpA2 and GbpA3, interact with the substrate-binding surface of the catalytic domain, as discussed in detail below. It is important to note that the remarkable difference between binding of the catalytic domain and the CBM73-truncated variant is only visible when the catalytic domain is reduced (compare Figure 2A with [Fig fig2]B), i.e., when it is catalytically competent and vulnerable to off-pathway reactions. Of note, X-ray and SAXS data for Cu­(II)-loaded *Vh*GbpA indicate an elongated shape,^50^ indicating that there are no domain interactions in the nonreduced enzyme.

### Activity and Stability during Chitin Degradation

Substrate-binding affinities of LPMOs can also be assessed by studying enzyme activity and stability in turnover conditions using varying substrate concentrations. Weak binding of the LPMO and/or low substrate concentrations will promote off-pathway reactions, which will lead to enzyme inactivation. In other words, an LPMO that binds its substrate weakly will perform less well at lower substrate concentrations compared to an LPMO that binds the substrate more strongly. It is important to note that in reductant-driven reactions, such as those discussed here, the rate of the reaction primarily depends on the rate of *in situ* generation of H_2_O_2_. Thus, the rate will not necessarily depend on enzyme affinity nor on the substrate concentration, even if this concentration is not saturating. Furthermore, inactivation of the LPMO will only become noticeable when the remaining fraction of active enzymes becomes too small to consume the *in situ* generated H_2_O_2_ productively.

In accordance with these latter considerations, an extensive study with the *Vh*GbpA variants using five different chitin concentrations showed that the initial rates of the chitin degradation reactions were similar for all three variants with only a modest dependency on the chitin concentration (Figure 3). However, the progress curves showed very different shapes. The full-length enzyme showed linear progress curves for the full 24 h of the reaction at all substrate concentrations, except the lowest (2.5 g·L^–1^) (Figure 3A). The catalytic domain alone only showed a linear progress curve at the highest tested chitin concentration (50 g/L) and showed increasingly fast deactivation as the substrate concentration became lower (Figure 3B). The CBM73-truncated variant showed clear signs of early inactivation at all tested substrate concentrations (Figure 3C). Due to fast inactivation, product levels in reactions with this latter enzyme remained low, at all tested substrate concentrations.

**Figure 3:**
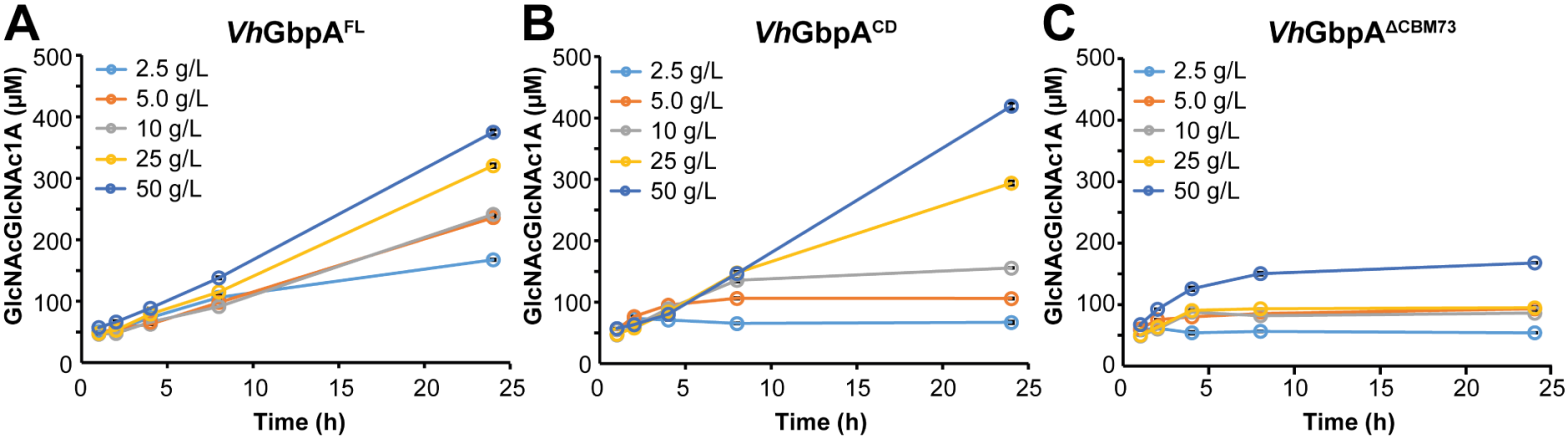
Reductant-driven chitin degradation by variants of *Vh*GbpA at varying substrate concentrations. The graphs show time courses for the formation of soluble oxidized products in reactions containing 0.5 μM *Vh*GbpA^FL^ (A), *Vh*GbpA^CD^ (B), or *Vh*GbpA^ΔCBM73^ (C) with β-chitin (2.5–50 g·L^–1^) in 20 mM Tris-HCl buffer, pH 7.5, fueled by 1 mM AscA. Through incubation with *Sm*CHB, soluble products were converted to a mixture of GlcNAc and chitobionic acid (GlcNAcGlcNAc1A) and the latter was quantified to determine the concentration of oxidized products. Each experiment was performed in triplicates; standard deviations were small and are hidden by the symbols.

The observed difference between *Vh*GbpA, with its CBM73, and *Vh*GbpA^CD^ is in line with the results of other studies on the effect of removing substrate-binding domains from two-domain LPMOs.^37,42^ Substrate binding through the CBM enhances the proximity of the catalytic domain to the substrate, increasing the chance that available H_2_O_2_ is used productively to oxidize the substrate rather than in a futile turnover reaction that may damage the enzyme. In light of existing literature data, the observed difference in activity and stability during chitin degradation between *Vh*GbpA^FL^ and *Vh*GbpA^CD^ is exactly what one would expect upon removal of the CBM.

The results obtained with *Vh*GbpA^ΔCBM73^ are puzzling but not surprising considering the binding studies described above. If this enzyme variant does not bind well to the substrate, as the binding studies suggest, and if this variant can still react with small molecules in off-pathway reactions, as the assessment of its redox properties suggests, one would expect the enzyme to rapidly inactivate in turnover conditions.

Another way to assess the ability of an LPMO to productively use H_2_O_2_ in a reaction with substrate is to look at progress curves for substrate turnover in reactions with varying amounts of exogenously added H_2_O_2_ at the start of the reaction. The higher the initial H_2_O_2_ concentration and the lower the ability of the LPMO domain to interact productively with the substrate, the faster enzyme inactivation will be observed. Progress curves obtained with different H_2_O_2_ concentrations (Figure 4) showed fast turnover of the substrate, in accord with the peroxygenase nature of these enzymes (note the minute time scale in Figure 4 compared to the hour time scale in Figure 3). In terms of tolerance to H_2_O_2_ and enzyme inactivation, the progress curves for both the *Vh*GbpA (Figure 4) and the *Vc*GbpA (Figure S6) variants show trends that align well with the trends observed for the reductant-driven reactions depicted in Figure 3.

**Figure 4:**
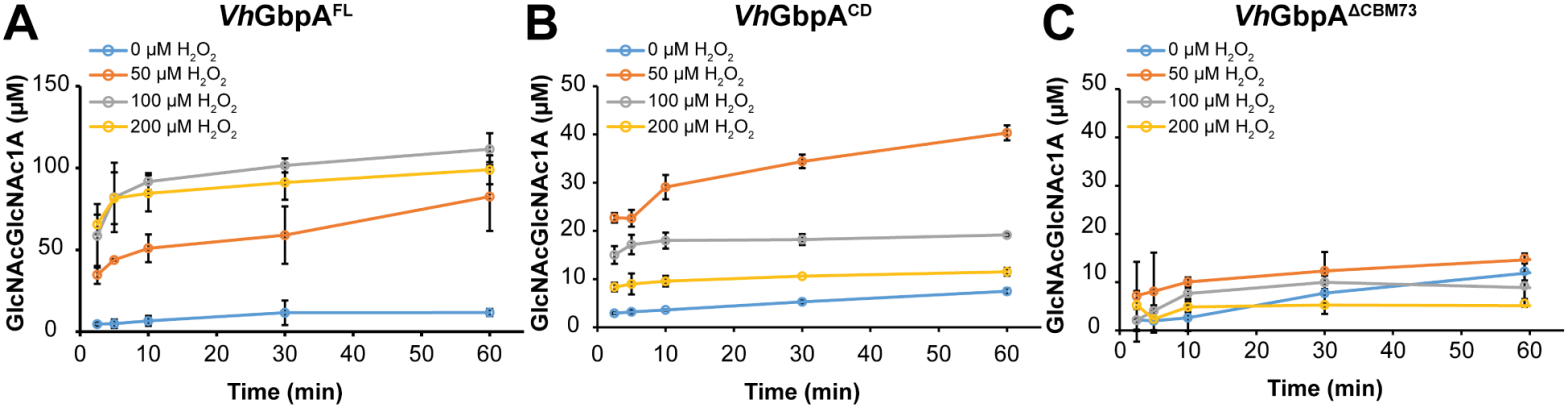
H_2_O_2_-driven degradation of chitin by *Vh*GbpA variants. The graphs show time courses for the formation of soluble oxidized products in reactions containing 0.5 μM *Vh*GbpA^FL^ (A), *Vh*GbpA^CD^ (B), or *Vh*GbpA^ΔCBM73^ (C), 10 g·L^–1^ β-chitin in 20 mM Tris-HCl, pH 7.5. After preincubating these mixtures at 30 °C with shaking at 1000 rpm for 30 min, reactions were initiated by sequentially adding H_2_O_2_ (to the indicated final concentrations) and, last, 0.1 mM AscA to start the reaction. Soluble oxidized products were quantified as described in the legend of Figure 3. All reactions were performed in triplicates, and the error bars show ± SD (*n* = 3). Note that product formation in the reaction with 0 mM H_2_O_2_ reflects reductant-driven LPMO activity; in this reaction, H_2_O_2_ is generated slowly, *in situ*. Identical experiments with the *Vc*GbpA variants gave similar results and are shown in Figure S6.

First, Figure 4 shows that the full-length enzyme is more capable of productively oxidizing chitin than the catalytic domain only. For the full-length enzyme, the reactions with 100 and 200 μM H_2_O_2_ both yielded about 100 μM of product, showing that notable inactivation only occurs at 200 μM H_2_O_2_. For the catalytic domain, product formation at 100 and 200 μM H_2_O_2_ amounted to approximately 20 and 10 μM, respectively, which is lower than the amount of product obtained in the reaction with 50 μM H_2_O_2_. This confirms the notion that the catalytic domain only, lacking the CBM, is less capable of productively using H_2_O_2_ and more prone to damaging off-pathway reactions. Second, Figure 4C shows that the CBM-truncated variant is rapidly inactivated at all H_2_O_2_ concentrations, confirming that the productive interaction of this enzyme with the substrate is severely hampered.

### Stability of the Reduced Enzyme in the Absence of Substrate

Given that reduced LPMOs are prone to oxidative self-inactivation and that the *Vh*GbpA variants show clear differences in catalytic performance, we assessed their stability in the absence of substrate using an ascorbic acid depletion assay (Figure 5). In this experiment, AscA depletion serves as a proxy for copper release following enzyme damage and inactivation, processes that are triggered under reductive, substrate-free conditions.^37,44^ In such conditions, the reduced LPMO will react with available H_2_O_2_ in a damaging off-pathway reaction and copper released from damaged active sites will promote oxidation (depletion) of AscA, increased production of H_2_O_2_, and further inactivation of the LPMO. Thus, the degree of AscA depletion reflects the extent to which the GbpA variants are protected from turning over H_2_O_2_ when there is no substrate present. The full-length enzyme showed the slowest consumption of AscA showing that it is the most stable and least likely to turn over H_2_O_2_ in the absence of substrate. The CBM73-truncated variant was somewhat less stable than the full-length enzyme, whereas, importantly, the catalytic domain, clearly was the least stable of the three GbpA variants. This shows that while the catalytic domain alone binds chitin and retains high activity, it is also more vulnerable to oxidative inactivation without the stabilizing contribution of the accessory domains GbpA2 and GbpA3. A plausible explanation for this observation, aligning with similar indications described above, would be that the GbpA2 and GbpA3 domains interact with the reduced copper site on the LPMO domain, thus reducing its tendency to turnover H_2_O_2_ in the absence of substrate.

**Figure 5:**
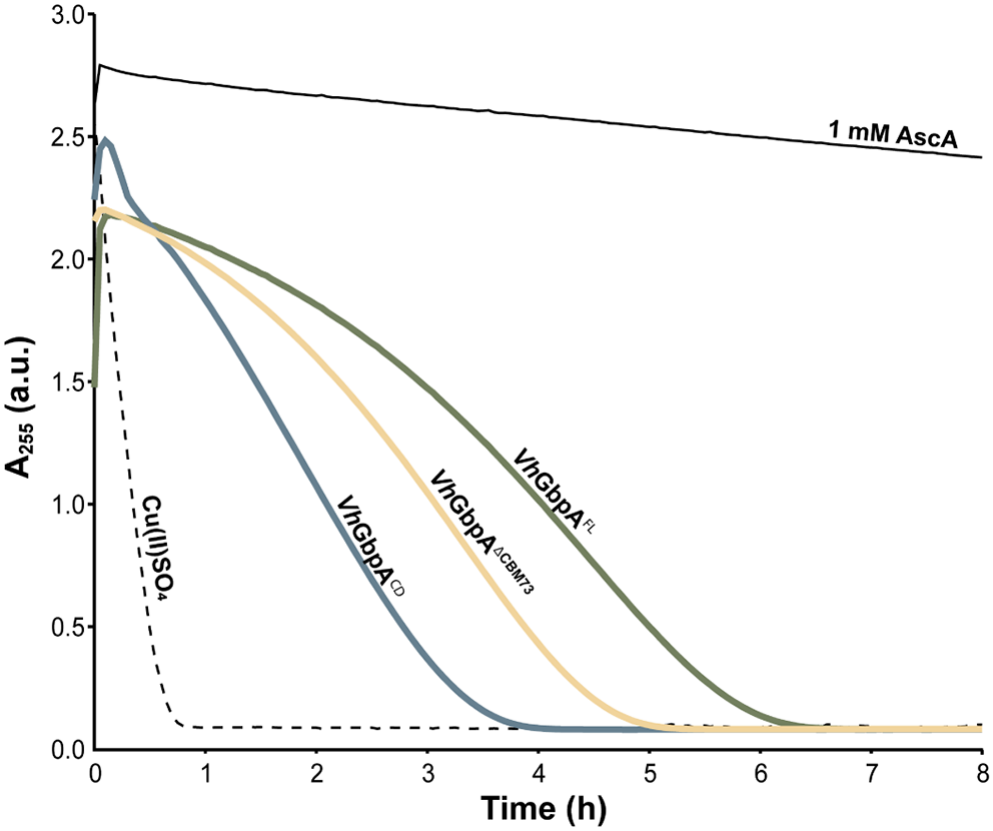
Ascorbic acid depletion in substrate-free reactions with *Vh*GbpA variants. Reactions containing 0.5 μM of each *Vh*GbpA variant were performed in 20 mM Tris-HCl buffer (pH 7.5) with 1 mM ascorbic acid (AscA). A control with 0.5 μM Cu­(II)­SO_4_ was included to monitor the effect of free copper, and an additional control lacking enzyme (1 mM AscA in buffer) was used to assess AscA stability over time. All reactions were carried out in technical triplicates (*n* = 3), but for clarity, only one representative curve per variant is shown, as replicates showed negligible variation. The mixtures were incubated at 30 for up to 8 h, and absorbance at 255 nm was recorded every 3 min. Gentle mixing was achieved through plate movement during measurements.

### An Interaction between GbpA3 and the Catalytic Domain

The crystal structures of CBM73-truncated *Vc*GbpA^45^ and of full-length *Vh*GbpA^50^ do not point at specific interactions between the GbpA2 or GbpA3 domains and the substrate-binding surface and catalytic center of the LPMO domain. SAXS studies of both enzymes suggest that they have an elongated form in solution.^45,50^ Nevertheless, the present data clearly indicate that when the CBM73 is truncated, the presence of the GbpA2 and GbpA3 domains hampers binding to chitin. Importantly, crystallization and SAXS studies of the GbpAs have been done using enzymes in their Cu­(II) state, whereas the findings described above refer to GbpAs in their Cu­(I) state. The AscA depletion assay, where the LPMO is in the Cu­(I) state, also suggests an interaction between the domains.

To gain more insight into these matters, we predicted the structures of both *apo*- and copper-loaded GbpAs using the AlphaFold3 online server.^55^ Most interestingly, the structural models predict that GbpA3 folds back onto the substrate-binding surface of the LPMO domain and that two surface-exposed histidines (His335 and His366 in *Vh*GbpA and His334 and His365 in *Vc*GbpA) in GbpA3 interact with the copper in the histidine brace (Figures 6 and S7). This interaction was only predicted when copper was included, and the absence or presence of the CBM73 did not change the outcome of these predictions. Of note, a recent preprint^68^ describes the same unique interdomain arrangement in AlphaFold3-generated models of a GbpA-like protein from the opportunistic pathogen *Enterobacter cloacae*. These structural predictions suggest a potential regulatory role for the GbpA3 domain in modulating access to the copper center of the LPMO domain, which aligns well with the experimental data described above.

**Figure 6:**
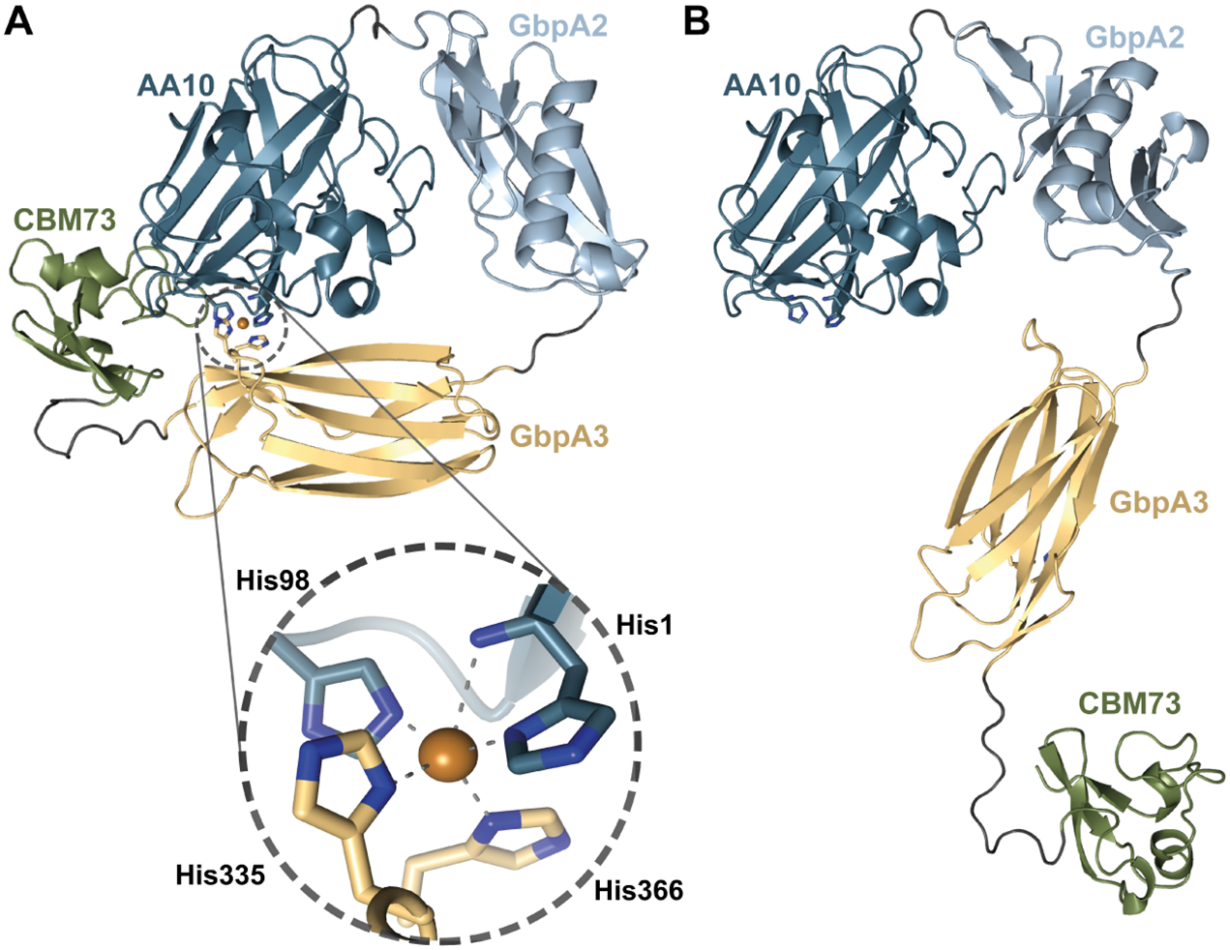
Cartoon representations of the structures of *holo* and *apo Vh*GbpA predicted by AlphaFold3. Panel A shows the copper-loaded (*holo*) *Vh*GbpA structure, where the GbpA3 domain folds over the AA10 catalytic site. In this conformation, two histidine residues from GbpA3 (His335 and His366) are positioned to coordinate the copper ion in the active site together with the histidine brace (His1 and His98). Panel B displays the *apo*-*Vh*GbpA structure, which adopts a more elongated conformation. A similar domain rearrangement upon copper binding is observed when comparing AlphaFold3 predictions for the structure of *apo* and *holo-Vc*GbpA (Figure S7).

An analysis of 165 GbpA-like protein sequences (InterPro IPR020879), each containing all four domains, revealed that the two histidines predicted to interact with the active site copper in *Vh*GbpA3 and *Vc*GbpA3 are strictly conserved across all sequences (Figure S8). Interestingly, for these 165 proteins, sequence conservation is markedly higher in the AA10 (LPMO) domain than in the GbpA3 domain (Figure S9). While the AA10 domain shows full conservation for 60 out of 182 positions, only 7 out of 89 residues in the GbpA3 domain are fully conserved and two of these are the histidines that are putatively interacting with the catalytic copper. An additional search in InterPro for proteins containing the GbpA3 domain led to the discovery of 28 multimodular GH18 chitinases harboring a GbpA3 domain, including a GH18 (UniProt ID A7MTY3) from *V. campbellii* ATCC BAA-1116, which is the source of the *Vh*GbpA studied here. Analysis of these non-LPMO GbpA3 domains revealed that the histidines conserved in GbpA-like LPMO proteins are absent. In fact, none of these two histidines were present in any of the GbpA3 domains of these GH18-containing proteins. The positions corresponding to His335 and His366 in *Vh*GbpA3 are instead occupied by a serine or an asparagine (His335 position) and a glutamine or a serine (His366 position), respectively. Thus, the presence of these two histidines in GbpA3 domains is correlated with the GbpA3 domain being part of an LPMO.

A further analysis of the interaction between the AA10 domain and the GbpA3 domain in *Vh*GbpA revealed highly conserved residues on the AA10 domain and the GbpA3 domain that could contribute to the interaction between these domains (Figure S10). For example, the side chain of Glu39 located on the substrate-binding surface of the LPMO domain can form a hydrogen bond with the side chain of Gln383 on the GbpA3 domain, while the main chain carbonyl of Gln383 can form a hydrogen bond with the side chain of Arg20. These residues are fully conserved, except the arginine at position 20, which is an exception in *Vh*GbpA; the other 164 GbpAs have a lysine, which can make a similar hydrogen bond (Figure S9). Finally, fully conserved Glu44 and Thr166 on the AA10 domain can form hydrogen bonds to the imidazole side chain of His335 (Figure S10A). Analysis of the LPMO-GbpA3 interface in the AlphaFold3 model of *Vh*GbpA with the PDBePISA server^69^ yielded a calculated interface area of 487 Å^2^, which is well compatible with interface areas typically observed for transient protein–protein interactions.^69^ This analysis identified 16 amino acids from the LPMO domain and 11 amino acids from the GbpA3 domain contributing to the interface (Figure S10B).

The combination of these structural predictions with the observation that chitin binding is most severely hampered by the GbpA3 domain in the reduced LPMO and, most remarkably, only in the absence of the CBM73, leads to a hypothesis for how the reactivity of the catalytically competent copper site may be modulated by the presence of substrate (Figure 7). One could envisage that in the absence of substrate the reduced copper in the catalytic LPMO domain is protected from engaging in off-pathway reactions by binding of GbpA3, as supported by, for example, the AscA depletion assay. Binding of the CBM73 to substrate could lead to elongation of the protein, pulling GbpA3 away from the LPMO domain, enabling the latter to interact with the substrate. In the CBM73-truncated variants, this “pulling effect” would not occur, explaining why these variants interact badly with chitin. In their recent preprint describing a GbpA-like protein from *E. cloacae*, Bardhan et al. propose a similar model based on AlphaFold3 models supported by studies of chitin binding and chitin degradation.^68^ Importantly, in the present study, we show that, indeed, the LPMO-GbpA3 interaction modifies the reactivity of the catalytic copper and the redox stability of the enzyme. We also show that this interaction is strongest when the copper is reduced, which makes sense from a functional perspective.

**Figure 7:**
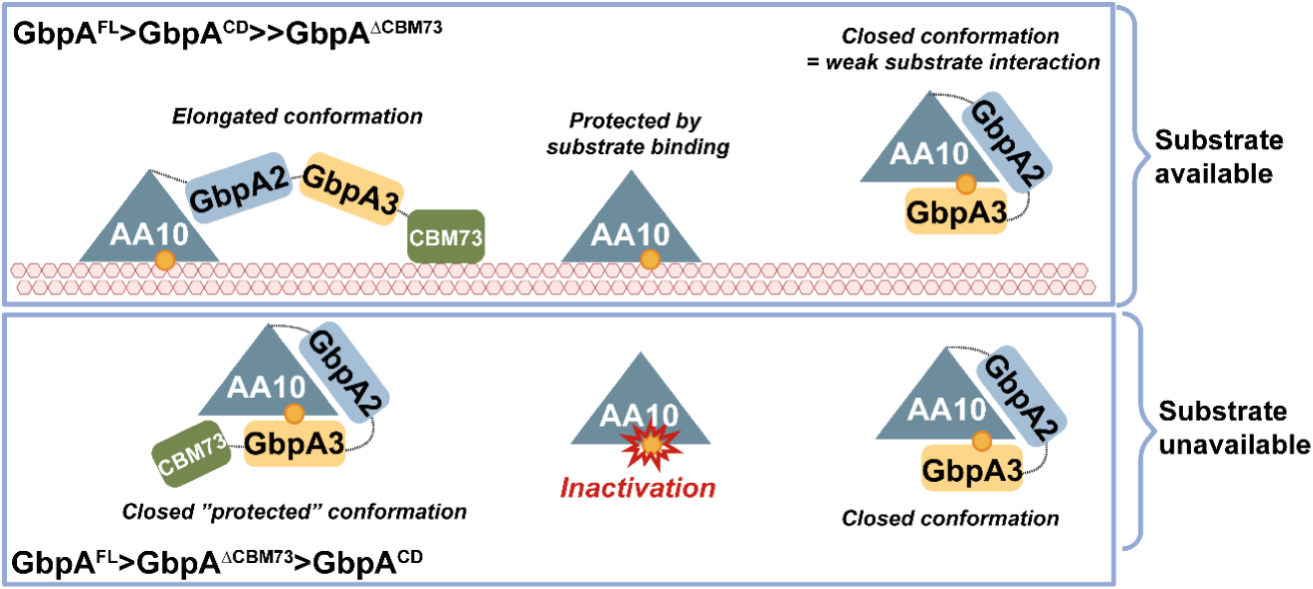
Proposed domain arrangement of GbpA in the presence and absence of chitin. When chitin is present, the catalytic AA10 domain and the CBM73 domain are predicted to bind the chitin surface, resulting in an elongated conformation that displaces the GbpA3 domain from the LPMO active site, thereby facilitating productive interactions between the LPMO and the substrate. In the absence of chitin, the model predicts that GbpA3 folds back over the substrate-binding surface of the LPMO. In this conformation, two surface-exposed histidines (His335 and His366 in *Vh*GbpA; His334 and His365 in *Vc*GbpA, see Figures 6 and S7) are positioned to interact with the catalytic copper ion within the histidine brace. In CBM73-truncated variants, the absence of both the CBM domain and substrate binding likely prevents the protein from adopting the elongated, active conformation, which explains their reduced ability to act on chitin and increased susceptibility to inactivation (relative to the other two enzyme variants in the presence of substrate). In the absence of substrate, the LPMO domain alone is more rapidly inactivated (indicated by the red symbol) than the CBM73-truncated variant because of the protective function of the GbpA3 domain in the latter. The order of the stabilities of the GbpA variants in the presence (upper panel) or absence (lower panel) of substrate is indicated in the figure.

Interestingly, a preprint by Sørensen et al.^70^ addresses the conformation of *Vc*GbpA when bound to chitin. Using negative-stain electron microscopy, Sørensen et al. conclude that *Vc*GbpA adopts an extended conformation when bound to chitin, well in line with the model proposed in Figure 7.

The interaction of the GbpA3 domain with the LPMO domain is strongly supported by the impact of GbpA3 on binding and degradation of chitin and by most of the additional functional analyses that assess copper reactivity in the absence of substrate. The observed higher oxidase activity for the catalytic domain alone compared to the two variants carrying the GbpA3 domain aligns very well with the hypothesis that GbpA3 affects the reactivity of the LPMO domain. However, single-turnover experiments for reduction by AscA and reoxidation by H_2_O_2_ did not show similarly large effects. It is conceivable that this reflects a limitation of the “single-turnover” setup, although the exact nature of such an effect remains elusive. While differences observed in the reduction and reoxidation rates were small (Table 1), these differences do in fact align with the rest of the data and our hypotheses: The LPMO domain alone shows a higher reoxidation rate, compared to the GbpA3-containing enzyme variants, which aligns with the notion that the LPMO is less protected in the absence of the GbpA3 domain. Finally, the AscA depletion assays support the notion that, in absence of substrate, the GbpA3 domain protects the copper from reacting with H_2_O_2_. Of note, this latter experiment may seem to suggest that the presence of the CBM73 somehow affects the LPMO-GbpA3 interaction, since *Vh*GbpA^FL^ and *Vh*GbpA^ΔCBM73^, both with the GbpA3 domain, show different depletion curves.

## Concluding Remarks

This study provides an in-depth functional characterization of an essential class of multidomain LPMOs with roles in bacterial virulence. While the LPMO domain of these GbpA proteins turns out to behave similarly to single-domain chitin-active LPMOs, the multidomain structure of these proteins sets them apart from LPMOs that seem to have evolved to degrade chitin, such as *Sm*AA10A from *S. marcescens*.^14,71^ It is worth noting that multidomain LPMOs associated with virulence occur in ecosystems where degradation of chitin does not seem to occur,^54^ and it may be questioned whether the generally observed activity on chitin for these proteins reflects their natural substrate preferences or whether these enzymes may have other, hitherto unknown substrates.

Our functional characterization, combined with structure predictions, clearly shows that the GbpA3 domain binds to the reduced LPMO domain. This provides a glimpse of the function of this domain, which we believe is linked to regulating the access to and reactivity of the copper site, as illustrated in Figure 7. This unique arrangement provides a novel concept in the LPMO field and may eventually contribute to understanding how these multidomain LPMO-containing virulence factors operate. We note that GbpA3-like proteins are widely distributed across bacterial species, with conserved sequence motifs suggesting functional relevance across diverse ecosystems.

It remains to be seen how this interaction mechanism plays out in natural environments. Does this protein primarily act on chitin, or are other substrates involved? Investigating CBM73 specificity and conducting *in vivo* experiments with truncation variants will be crucial to resolving these questions. Furthermore, investigating if and how GbpA2 and GbpA3 interact with chitin surfaces, or potentially other biomolecular structures, could provide broader insights into their roles in substrate recognition, enzymatic efficiency, and into their ecological relevance. The potential impact of the CBM73 on the overall domain arrangement also needs further attention, since the ascorbate depletion assay, unexpectedly, showed that deletion of the CBM affects stability. Of note, in their preprint describing studies of the GbpA-like protein of *E. cloacae*, Bardhan et al., 2025 conclude that the GbpA2 and GbpA3 domains do not bind to chitin at physiological pH.^68^

To better understand the interaction between GbpA3 and the LPMO domain, as well as the functional implications of this interaction, additional work is needed. This could include affinity studies using isolated domains, site-directed mutagenesis, and investigations into conformational changes upon substrate binding. It would also be of significant interest to study how this newly discovered domain–domain interaction affects GbpA functionality *in vivo*. Despite being around for two decades now, many questions remain regarding the *in vivo* functions of these intriguing virulence factors. The present results suggest that the unique domain arrangement in GbpA-like proteins not only relates to substrate binding but also to protecting the enzyme from damaging off-pathway reactions.

## Materials and Methods

### Materials

All chemicals were obtained from Sigma-Aldrich (St. Louis, MO, USA) unless specified otherwise. The model microcrystalline chitin substrate used in the study is commercial β-chitin from squid pen (5 mm flakes) acquired from Glentham Life Science Ltd. (Corsham, UK), which was milled and sieved in-house to a particle size of <75 μm using a PM200 planetary ball mill (Retsch, Haan, Germany) with zirconium oxide grinding tools. 100 mM ascorbic acid stock solutions were prepared in metal-free TraceSELECT water (Honeywell, Charlotte, NC, USA), filter-sterilized using 0.22 μm syringe filters, and aliquoted and stored at −20 °C in light-protected tubes, and were used only once after thawing. Tryptone and yeast extract were obtained from Thermo Fisher Scientific.

### Protein Production and Purification

The gene coding for full-length *Vh*GbpA^FL^ (amino acid residues 24–487, AA10-GbpA2-GbpA3-CBM73, accession number ABU72648) was synthesized by GenScript Biotech (Piscataway, NJ, USA) as described previously,^50^ while the genes encoding the two truncated variants, the LPMO catalytic domain alone (*Vh*GbpA^CD^, amino acid residues 24–201) and the CBM73 truncated variant (*Vh*GbpA^ΔCBM73^, amino acid residues 24–415) were synthesized after codon optimization for *E. coli* expression by Synbio Technologies (NJ, USA). The genes were cloned into pET-20b­(+) for *Vh*GbpA^CD^ or pET-22b­(+) for *Vh*GbpA^ΔCBM73^, using NdeI/XhoI restriction sites, leading to in-frame fusions with nucleotide sequences encoding the 22-amino-acid *pelB* signal peptide at the N-terminus, to facilitate protein export to the periplasm, and six histidine residues at the C-terminus.

Wild-type *Vh*GbpA^FL^ was expressed and purified as described previously.^50^ To produce the truncated forms, the plasmids were transformed by heat shock into chemically competent One Shot BL21 Star (DE3) cells (Invitrogen). Fresh colonies were inoculated in Luria–Bertani broth (LB medium) supplied with 100 μg·mL^–1^ ampicillin. To induce protein expression, the bacteria were grown in LB medium (37 °C, 220 rpm) to an OD_600_ of 0.6–0.8 before induction with isopropyl-β-d-thiogalactoside (IPTG) at a final concentration of 0.2 mM and then grown at 20 °C, 200 rpm for 16 h. Cells were harvested by centrifugation (8000 × g for 10 min at 4 °C) using a Beckman Coulter centrifuge (Brea, CA, USA), after which periplasmic proteins were extracted using cold osmotic shock with 1.0 mM of magnesium chloride, as previously described.^72^ The resulting periplasmic fractions were sterilized by filtration through a 0.22 μm syringe filter prior to storage at 4 °C and subsequent purification of the enzyme. The extracts were subjected to anion exchange chromatography using a 5 mL HiTrap HP (Q Sepharose) column (GE Healthcare, Chicago, USA). After the application of up to 30 mL sample, the column was washed with 10 column volumes of 20 mM Tris–HCl, pH 7.5, followed by elution with a linear gradient of NaCl (0–1 M over ten column volumes) in 20 mM Tris–HCl, pH 7.5, at a flow rate of 2 mL·min^–1^. LPMO-containing fractions were identified by SDS-PAGE, pooled, concentrated to approximately 2 mL using Vivaspin ultrafiltration tubes, 10 kDa MWCO (Sartorius, Göttingen, Germany) and loaded onto a ProteoSEC Dynamic 16/60 3–70 HR preparative size-exclusion column (Protein Ark, Sheffield, UK) operated at 1 mL·min^–1^ flow rate and equilibrated with 20 mM Tris-HCl, pH 7.5. The purity of resulting protein preparations was confirmed by SDS-PAGE. Protein concentrations were determined using UV spectroscopy at 280 nm and theoretical extinction coefficients, which were predicted using the Expasy ProtParam tool.

*Vc*GbpA^FL^ and its truncated variants, *Vc*GbpA^CD^ and *Vc*GbpA^ΔCBM73^, were produced and purified as previously reported, without a His-tag on the C-terminus.^45^ The GH20 β-N-acetylhexosaminidase, or chitobiase, from *Serratia marcescens*, here referred to as *Sm*CHB, was recombinantly produced and purified as previously described.^52^ The GH18 chitinases A and C from *Serratia marcescens* (*Sm*Chi18A and *Sm*Chi18C) were recombinantly produced as described previously.^73^

### Copper Saturation

Solutions with purified LPMOs (in 20 mM Tris-HCl, pH 7.5) were concentrated to reach a total volume of about 2 mL using Vivaspin ultrafiltration tubes (10 kDa MWCO; Sartorius, Göttingen, Germany), after which they were supplemented with a 2-fold molar surplus of Cu­(II)­SO_4_, followed by incubation at room temperature for 30 min. In some cases, this was done prior to the size-exclusion chromatography step of the purification protocol, which subsequently removed excess copper. To ensure the removal of free copper in protein samples treated after size exclusion chromatography, the samples were subjected to multiple consecutive rounds of concentration and dilution in 20 mM Tris-HCl, pH 7.5, using Amicon Ultra-15 Centrifugal filter units (Merck, Darmstadt, Germany). The resulting total dilution factor for solutions of copper-saturated LPMOs amounted to at least 1,000,000-fold. Purified enzymes were stored at 4 °C until use.

### Reduction and Reoxidation Kinetics

Single turnover experiments to determine the rate of the reduction of LPMO-Cu­(II) to LPMO-Cu­(I) by ascorbate were performed using a single-mixing setup, while the reoxidation of LPMO-Cu­(I) to LPMO-Cu­(II) by H_2_O_2_ was assessed using a double-mixing setup, essentially as described previously using the fluorescence change that accompanies reduction or oxidation of the LPMO for detection.^58,59^ All experiments were conducted with an SFM-4000 stopped-flow system equipped with a MOS-200 M dual absorbance fluorescence spectrometer (BioLogic, Grenoble, France), with an applied voltage of 600 mV for detection. The excitation wavelength was set to 280 nm, and the increase (for reduction) or decay (for reoxidation) of fluorescence intensity was collected with a 340 nm bandpass filter. All experiments were carried out at 25 °C in 20 mM Tris-HCl, pH 7.5. Anaerobic conditions were obtained by storing N_2_–purged buffers and labware in a Whitley A95TG anaerobic workstation (Don Whitley Scientific, West-Yorkshire, UK) for 24 h prior to preparing the necessary solutions in the anaerobic workstation. Purging with N_2_ was achieved using a Schlenk line, and all solutions were eventually transferred to sealed syringes in the anaerobic chamber.

To determine LPMO reduction rates, 75 μL of a 10 μM LPMO-Cu­(II) solution (5 μM as final concentration) was mixed with 75 μL of an ascorbic acid solution with concentrations ranging from 50 to 800 μM (25–400 μM after mixing) at 25 °C, ensuring pseudo-first-order conditions ([E] ≪ [S]). For reoxidation, double-mixing experiments were performed in two steps. In the first step, the LPMO-Cu­(II) (10 μM initial concentration) was mixed with one molar equivalent of l-cysteine for 10 s to form LPMO-Cu­(I). In a second step, the *in situ* generated LPMO-Cu­(I) was mixed with different concentrations of H_2_O_2_ (ranging from 12.5 to 800 μM after mixing), followed by monitoring the decay of fluorescence intensity. The stopped-flow rapid spectrophotometer was flushed with a significant excess of deoxygenated buffer before connecting the anaerobically prepared syringes containing the solutions to be used and performing the experiments.

Pseudo-first-order reaction rates (*k*_obs_) were determined by solving a single exponential equation with correction of baseline drift using the following equation: $y=at+b+ce^{-k_{obs}t}$ . In all cases, plots of *k*_obs_ vs [ascorbate] or [H_2_O_2_] were fitted using linear least-squares regression to obtain the apparent second-order rate constant for the reduction or reoxidation step (*k*_AscA_ or *k*_H_2_O_2__, respectively). All experiments were done at least in triplicates.

For *Vh*GbpA^FL^, reoxidation rates using the stopped-flow method could not be determined due to baseline drift of the reoxidized LPMO-Cu­(II). Therefore, fluorescence spectroscopy was employed to investigate this reoxidation rate. The LPMO-Cu­(II) (2 μM in a 1 mL fluorescence quartz cuvette) was initially mixed by stirring (sealed by parafilm M) with one molar equivalent of l-cysteine for 120 s to form LPMO-Cu­(I), after which the generated LPMO-Cu­(I) was mixed with different concentrations of H_2_O_2_ (ranging from 2 to 20 μM after mixing) followed by monitoring the decay of fluorescence intensity.

The reoxidation of LPMO-Cu­(I) by O_2_ under ambient conditions (i.e., an O_2_ concentration of approximately 250 μM) was monitored by measuring the decay of fluorescence intensity, as previously reported, with minor modifications.^59^ 2 μM LPMO-Cu­(II) was initially mixed with an equimolar amount of l-cysteine for 60 s to generate LPMO-Cu­(I). The generated LPMO-Cu­(I) was subsequently reoxidized by O_2_ in the solution. The decay of fluorescence intensity (excitation, 280 nm; emission, 342 nm) of LPMO-Cu­(I) was recorded over the course of 1 h. Each experiment was performed in duplicates.

### Substrate Binding

To examine the impact of the CBM73 domain on chitin-binding, *Vh*GbpA variants [i.e., *Vh*GbpA^FL^ (10 μM), *Vh*GbpA^CD^ (20 μM), or *Vh*GbpA^ΔCBM73^ (10 μM)] were incubated with 10 g·L^–1^ of β-chitin in 20 mM Tris-HCl (pH 7.5) at 22 °C and 1000 rpm for 1 h, either in the absence or presence of 1 mM AscA, in a final volume of 200 μL. The binding time was counted from the moment ascorbate was added (or buffer, in the reactions without AscA), while a reaction without substrate was included as a control. The binding event was stopped by centrifuging at 22 °C and 13,000 rpm for 10 min. The supernatant was collected for further analysis. The pellet was resuspended and washed with 200 μL of the same buffer, and this washing step was repeated twice. The supernatant from the second-round washing was kept for analysis, while the pellet was resuspended in 200 μL SDS loading buffer and heated at 95 °C for 10 min. All fractions were then analyzed by SDS-PAGE, with sample volumes adjusted to represent the same proportion of the original reaction mixture.

Binding to β-chitin was also evaluated with the same time-course reaction conditions as for chitin degradation (see below). Reactions were performed at 22 °C using reaction mixtures containing 2 μM enzyme and 10 g·L^–1^ of β-chitin in 20 mM Tris-HCl, pH 7.5. Ascorbate was added to a final concentration of 1 mM and was the last reagent to be added. Samples were taken after 1, 3, 5, 10, 30, and 60 min of incubation and separated from the insoluble chitin by filtration using a 96-well filter plate (Merck) operated with a vacuum manifold (Millipore). The unbound protein in the filtrate was quantified with an adapted Bradford protocol for measuring low protein concentrations.^67,74^ For each *Vh*GbpA variant, a control reaction without β-chitin was included and used as a reference to calculate the fraction (%) of unbound protein. Controls without enzymes were also added to monitor unspecific signals in the Bradford assay. All reactions were done in triplicates.

### Degradation of Chitin

Reductant-driven chitin-degradation experiments (“monooxygenase conditions”) were carried out in 2 mL Eppendorf tubes in an Eppendorf Thermomixer C (Eppendorf, Hamburg, Germany) set to 30 °C and 1000 rpm. If not stated otherwise, 0.5 μM LPMO was preincubated with 10 g·L^–1^ squid pen β-chitin (Glentham Life Science Ltd., Corsham, UK) with a particle size of <75 μm for 30 min at 30 °C before adding 1 mM AscA to start the reaction. All LPMO reactions were carried out in 20 mM Tris-HCl, pH 7.5.

The reactions were sampled at various time points, and enzyme activity in the samples was stopped by separating the LPMO from the insoluble substrate using a MultiScreen 96-well filter plate (Millipore, Burlington, MA, USA) operated with a Millipore vacuum manifold. Qualitative analysis of soluble oxidized products was carried out by matrix-assisted laser desorption/ionization-time-of-flight mass spectrometry, MALDI-ToF MS, using a Bruker Autoflex mass spectrometer equipped with a 337 nm nitrogen laser. 1 μL of the reaction mixture was mixed with 1 μL of 2,5-dihydroxybenzoic acid (10 g·L^–1^ dissolved in 30% acetonitrile with 0.1% (v/v) trifluoroacetic acid) and applied onto an MTP 384 target plate ground steel TF (Bruker Daltonics GmbH, Bremen, Germany), followed by drying under a stream of air. Data acquisition and analysis were performed using FlexControl and FlexAnalysis (Bruker Daltonics GmbH).

For quantification of soluble oxidized chitooligosaccharides generated by the LPMO, the filtrates were transferred to new tubes and supplemented with *Sm*CHB to a final concentration of 0.5 μM, followed by static incubation at 37 °C overnight. Treatment with *Sm*CHB converts longer native and oxidized chitooligomers to a mixture of *N*-acetylglucosamine (GlcNAc, native monomer) and oxidized chitobiose (GlcNAcGlcNAc1A, oxidized dimer), which simplifies quantification of soluble reaction products, as described previously.^52^

Reactions with exogenously added H_2_O_2_ were set up in the same manner. After the 30 min preincubation, varying concentrations of H_2_O_2_ (0, 50, 100, and 200 μM) were added after which the reaction was initiated by addition of AscA to a final concentration of 0.1 mM. Sampling, sample treatment with *Sm*CHB and product analysis were performed as described above.

### Quantification of Reaction Products

Quantification of chitobionic acid (GlcNAcGlcNAc1A) and *N*-acetylglucosamine (GlcNAc) was done by chromatography using an RSLC system (Dionex, Sunnyvale, CA, USA) equipped with a 100 × 7.8 mm Rezex RFQ-Fast Acid H^+^ (8%) (Phenomenex, Torrance, CA, USA) column operated at 85 °C. Samples of 8 μL were injected into the column, and sugars were eluted isocratically for 6 min with 5 mM sulfuric acid as mobile phase and a flow rate of 1 mL·min^–1^. The analytes were monitored using a 194 nm UV detector. Standards of GlcNAcGlcNAc1A (25–800 μM) and GlcNAc (50–2500 μM) were used for quantification. GlcNAc was purchased from Megazyme (Bray, Ireland; 95% purity), while GlcNAcGlcNAc1A was generated in-house by complete oxidation of *N*-acetyl-chitobiose (Megazyme, Bray, Ireland; 95% purity) with a chitooligosaccharide oxidase from *Fusarium graminearum*,^75^ as previously described.^52^

### Oxidase Activity

The oxidase activity of the various GbpA variants was measured using the protocol described by Kittl et al.^56^ with modifications. Briefly, 50 μL of a reaction premixture comprised of 0.2 mM Amplex Red and 10 U·mL^–1^ horseradish oxidase (HRP) in Tris-HCl buffer (20 mM, pH 7.5) was mixed with 40 μL LPMO (1 μM as the final concentration in the reaction) prepared in the same buffer, followed by incubation at 30 °C for 5 min. 10 μL of 10 mM AscA was added to start the reaction. The microtiter plate was shaken for 30 s at 600 rpm prior to recording absorbance at 563 nm every 30 s for 60 min. Control reactions without enzyme and with free copper instead of enzyme were included. The amount of H_2_O_2_ produced was quantified based on an H_2_O_2_ standard curve (0 to 20 μM), including 1 mM AscA. All reactions were performed in triplicates.

### Determination of Apparent Melting Temperature (*T*_m_)

The apparent melting temperature (*T*_m_) of *Vh*GbpA variants was determined using the protein thermal shift assay (Thermo Fisher Scientific, Waltham, MA, USA). This assay employs the fluorescent dye SYPRO Orange to monitor protein unfolding.^76^ The quantum yield of SYPRO Orange increases significantly upon binding to hydrophobic regions that become exposed during protein unfolding. The fluorescence emission was monitored using a StepOnePlus real-time PCR machine (Thermo Fisher Scientific). For the assay, 5 μM enzyme was prepared in 20 mM Tris-HCl buffer (pH 7.5), with or without 5 mM EDTA, and incubated with SYPRO Orange dye in a 96-well plate (MicroAmp Fast Optical 96-well reaction plate with barcode 0.1 mL, Thermo Fisher Scientific) covered by an adhesive film (MicroAmp Optical Adhesive Film, Thermo Fisher Scientific). The temperature was gradually increased from 25 to 99 °C over a duration of 75 min. Each experiment was carried out in triplicate (*n* = 3) to ensure reproducibility.

### Ascorbic Acid Depletion

Reactions containing 0.5 μM of *Vh*GbpA variants and 1 mM ascorbic acid (AscA) were prepared by mixing equal volumes (50 μL each) of protein solution (1 μM) and AscA solution (2 mM) directly in a 96-well UV-transparent plate (Corning, Corning, NY, USA). AscA depletion was monitored spectrophotometrically by measuring absorbance at 255 nm every 3 min for up to 8 h using a Varioskan LUX plate reader (Thermo Fisher Scientific, Waltham, MA, USA). All experiments were performed in triplicates (*n* = 3).

## Supplementary Material



## Notes

Thermal shift assays; MALDI-ToF MS spectra of LPMO reaction products; reduction and reoxidation kinetics; H_2_O_2_-driven chitin degradation by *Vc*GbpA variants; AlphaFold3-predicted structures of *apo*- and *holo*-GbpA variants; amino acid frequency logos (WebLogos) of GbpA3 and GbpA-like AA10s; predicted interactions between GbpA3 and the AA10 domain with interface area calculations (PDBePISA); and oxidase rates for all LPMO variants (PDF)
