# Supplementary material for: Functional Characterization of Multidomain LPMOs from Marine Vibrio Species Reveals Modulation of Enzyme Activity by Domain–Domain Interactions
Source: Biochemistry. 2025 Dec 12;65(1):90–103. doi: 10.1021/acs.biochem.5c00529 (PMC12798814; doi:10.1021/acs.biochem.5c00529)
Supplement: Supplementary file 1 [file bi5c00529_si_001.pdf]

Supplementary Information for

Functional Characterization of Multi-Domain LPMOs from Marine *Vibrio* Species Reveals Modulation of Enzyme Activity by Domain-Domain Interactions

Yong Zhou[1,2,3], Eirik G. Kommedal[2], Zarah Forsberg[2], Gustav Vaaje-Kolstad[2], Wipa Suginta[1], and Vincent G. H. Eijsink[2,*]

[1] School of Biomolecular Science and Engineering (BSE), Vidyasirimedhi Institute of Science and Technology (VISTEC), Rayong 21210, Thailand.

[2] Faculty of Chemistry, Biotechnology and Food Science (KBM), Norwegian University of Life Sciences (NMBU), Ås 1432, Norway.

[3]Agricultural Genomics Institute at Shenzhen (AGIS), Chinese Academy of Agricultural Sciences (CAAS), Shenzhen 518120, China

*Correspondence to Vincent G.H. Eijsink, E-mail: vincent.eijsink@nmbu.no.

This PDF file includes:

1. List of Supplementary Figures and Table pg. S2
2. Supplementary Figures (1-10) pg. S3-S13
3. Supplementary Table 1 pg. S14
4. Supplementary References pg. S15

1. List of Supplementary Figures and Table

Figure S1. Thermal unfolding of *Vh*GbpA variants.

Figure S2. Oxidized products generated by *Vh*GbpA variants reacting with chitin.

Figure S3. Oxidized products generated by *Vc*GbpA variants reacting with chitin.

Figure S4. Reduction and reoxidation rates of *Vh*GbpA and *Vc*GbpA variants.

Figure S5. Reoxidation by O_2.

Figure S6. H_2O_2-driven degradation of chitin by *Vc*GbpA variants.

Figure S7. Cartoon representations of *apo*- and *holo*-GbpA structures predicted using AlphaFold3.

Figure S8. Amino acid frequency per position based on a multiple sequence alignment of 165 sequences encoding GbpA3 domains associated with LPMOs.

Figure S9. Amino acid frequency per position in the AA10 domains of 165 GbpA-like sequences.

Figure S10. Possible interactions between the GbpA3 domain and the AA10 domain in *holo*-*Vh*GbpA.

Table S1. The oxidase rate of LPMO variants.

2. Supplementary Figures (1-10)

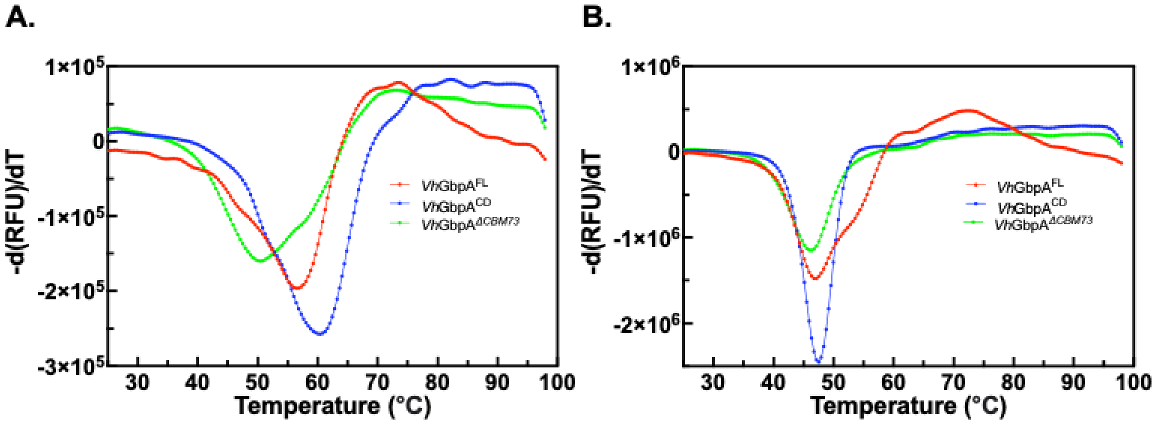

	**Apparent T_m (°C)**	
	Without 5 mM EDTA	With 5 mM EDTA
*Vh*GbpA^{FL}	56.4 ± 0.0	47.1 ± 0.1
*Vh*GbpA^{CD}	60.4 ± 0.1	47.5 ± 0.1
*Vh*LGbpA$^{\Delta CBM73}$	50.4 ± 0.0	46.3 ± 0.0

Figure S1. Thermal unfolding of *Vh*GbpA variants. The reaction mixtures contained 5 µM enzyme and SYPRO Orange dye and were incubated in the absence (A) or presence (B) of 5 mM EDTA in 20 mM Tris-HCl buffer (pH 7.5). The temperature was gradually increased from 25°C to 99°C over 75 minutes. Each experiment was carried out in triplicate ($n = 3$) to ensure reproducibility. The table below the graphs presents the apparent melting temperatures (T_m) derived from the unfolding curves.

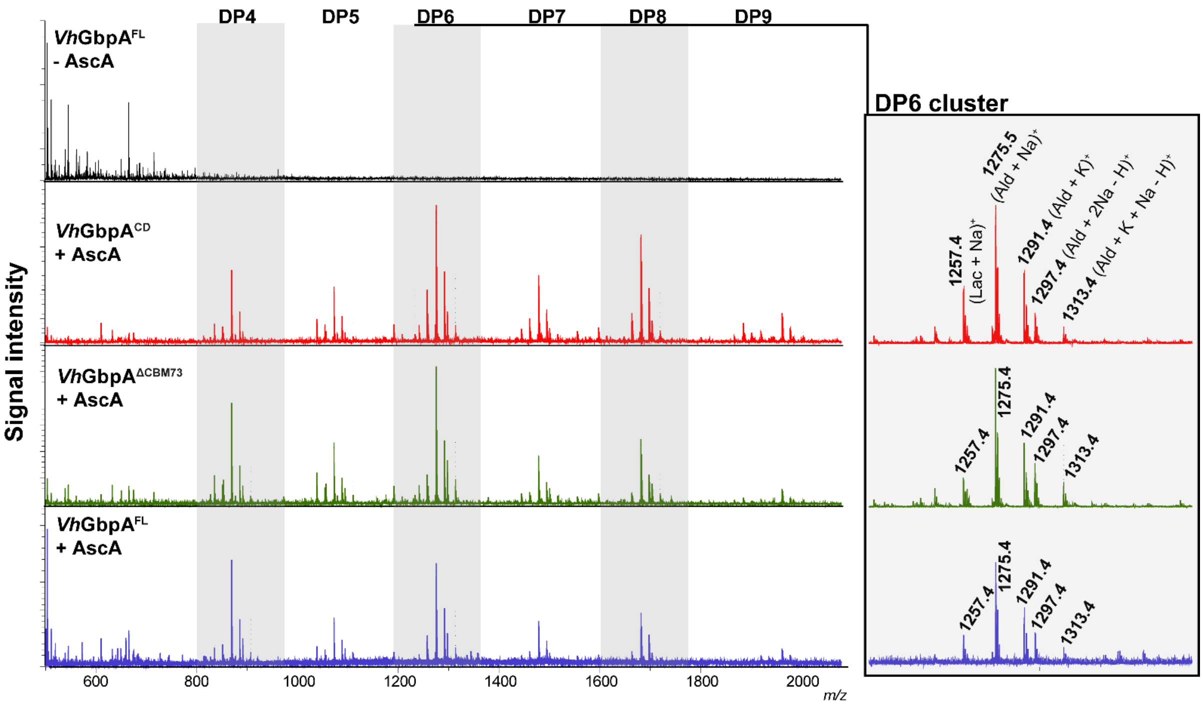

Figure S2. Oxidized products generated by *Vh*GbpA variants reacting with chitin. Reactions were performed with 0.5µM LPMO and 10 g.L^{-1} β-chitin in 20 mM Tris–HCl buffer (pH 7.5) containing 1 mM AscA, at 30°C, with shaking at 1000 rpm, for 24 hours. The reactions were stopped by filtration using a MultiScreen™ 96-well filter plate operated with a Millipore vacuum manifold. Product mixtures were subsequently analyzed using MALDI-TOF MS. The left panel shows raw spectra of the reaction mixtures (blue, *Vh*GbpA^{FL}; red, *Vh*GbpA^{CD}; green, *Vh*GbpA^{ΔCBM73}), whereas the right panel provides a zoomed-in view of the signals corresponding to hexameric products, with annotation of multiple signals. A control reaction with *Vh*GbpA^{FL} but without AscA was included, represented by the top black spectrum in the left panel.

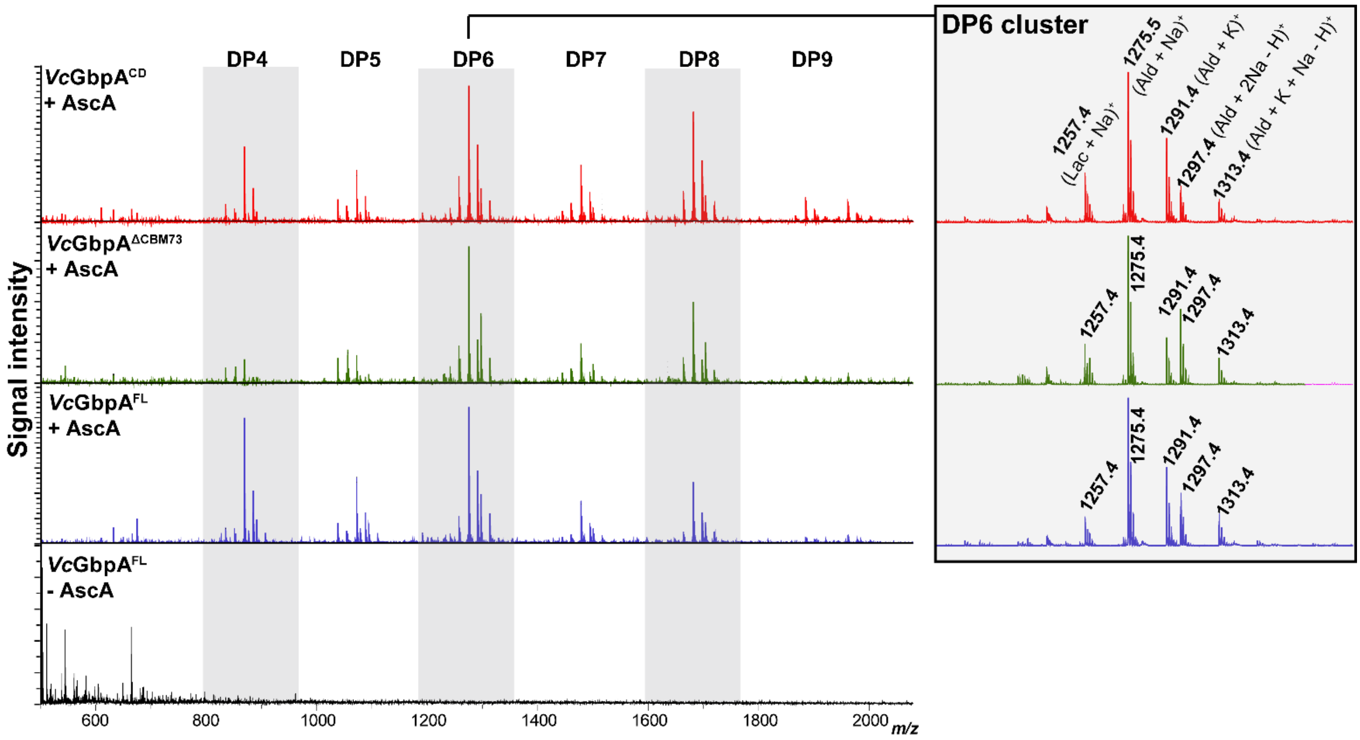

Figure S3. Oxidized products generated by *Vc*GbpA variants reacting with chitin. Reactions were performed with 0.5µM LPMO and 10 g.L^{-1} β-chitin in 20 mM Tris–HCl buffer (pH 7.5) containing 1 mM AscA, at 30°C, with shaking at 1 000 rpm, for 24 hours. The reactions were stopped by filtration using a MultiScreen™ 96-well filter plate operated with a Millipore vacuum manifold. Product mixtures were subsequently analyzed using MALDI-TOF MS. The left panel shows raw spectra of the reaction mixtures(blue, *Vc*GbpA^{FL}; red, *Vc*GbpA^{CD}; green, *Vc*GbpA$^{\Delta CBM73}$), whereas the right panel provides a zoomed-in view of the signals corresponding to hexameric products, with annotation of multiple signals. A control reaction with *Vc*GbpA^{FL} but without AscA was included, represented by the bottom black spectrum in the left panel.

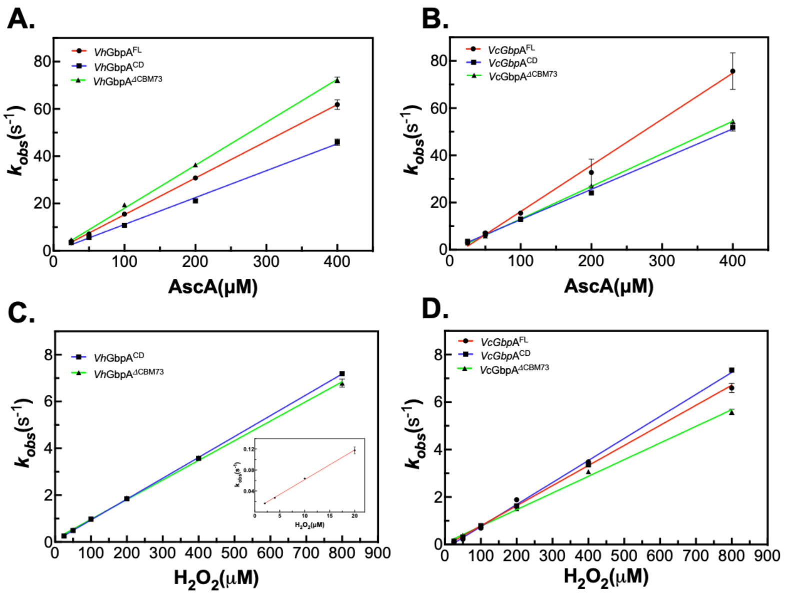

Figure S4. Reduction and reoxidation rates of *Vh*GbpA and *Vc*GbpA variants. A-D. Pseudo-first-order rate constants, k_{obs}, obtained in 20 mM Tris-HCl, pH 7.5, at 25°C, are plotted as a function of the concentration of AscA (reduction; panels **A, B**) or H_2O_2 (reoxidation; panels **C, D**). For *Vh*GbpA^{FL}, reoxidation could not be determined using stopped-flow fluorometry and was therefore measured by fluorescence spectroscopy instead (inset in panel **C**). Lower (2-20 μM) H_2O_2 concentrations were used because the manual setup does not allow immediate measurement after mixing, unlike the rapid mixing capability of stopped-flow, which is essential when using high H_2O_2 concentrations due to the fast reoxidation reaction. Each experiment was performed in triplicates and the error bars show ± S.D. (n = 3). The second order rate-constants derived from these plots are presented in **Table 1** in the main text.

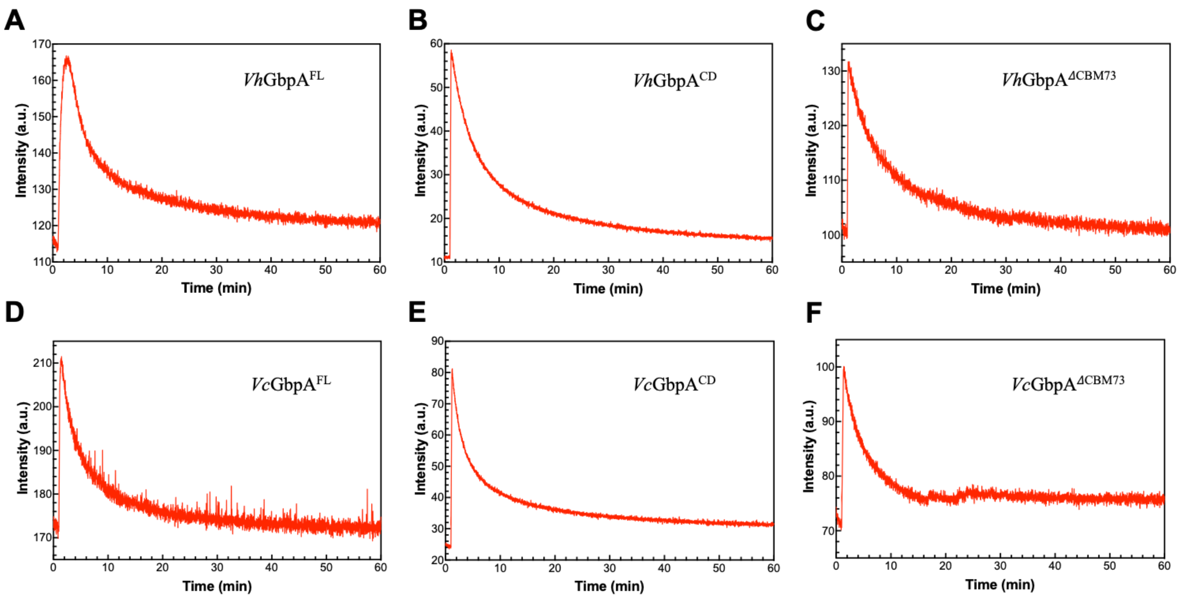

Figure S5. Reoxidation by O_2. Reaction mixtures contained 2 μM enzyme in 20 mM Tris-HCl, pH 7.5, and were kept at 25 °C. Reduction of *Vh*GbpA^{FL} (**A**), *Vh*GbpA^{CD} (**B**), *Vh*GbpA$^{\Delta CBM73}$ (**C**), *Vc*GbpA^{FL} (**D**), *Vc*GbpA^{CD} (**E**) and *Vc*GbpA$^{\Delta CBM73}$ (**F**) was achieved by the addition of 2 μM L-cysteine after approximately 1 minute, and is reflected by an increase in the fluorescence intensity ($\lambda_{Ex/Em}$ = 280/342 nm).

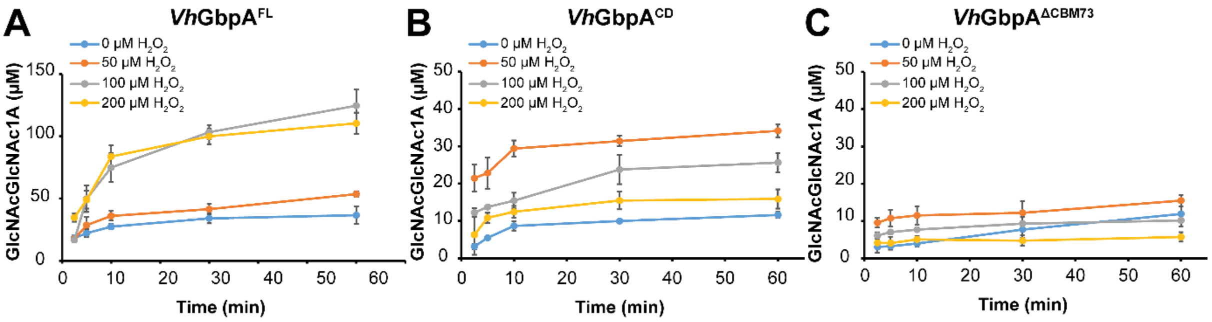

Figure S6. H_2O_2-driven degradation of chitin by *Vc*GbpA variants. The graphs show time courses for the formation of soluble oxidized products in reactions containing 0.5 µM *Vc*GbpA^{FL} (**A**), *Vc*GbpA^{CD} (**B**) or *Vc*GbpA$^{\Delta CBM73}$(**C**), and 10 g.L^{-1} β-chitin in 20 mM Tris-HCl, pH 7.5. After pre-incubating these mixtures at 30°C with shaking at 1,000 rpm for 30 mins, the reactions were initiated by sequentially adding H_2O_2 (to the indicated final concentrations) and, lastly, 0.1 mM AscA to start the reaction. Soluble oxidized products were quantified as described in the legend of **Fig. 3** (only chitobionic acid is shown). All reactions were performed in triplicates and the error bars show ± S.D. (n = 3). Note that product formation in the reaction with 0 mM H_2O_2 reflects reductant-driven enzyme activity; in these reactions H_2O_2 is generated slowly, *in situ*.

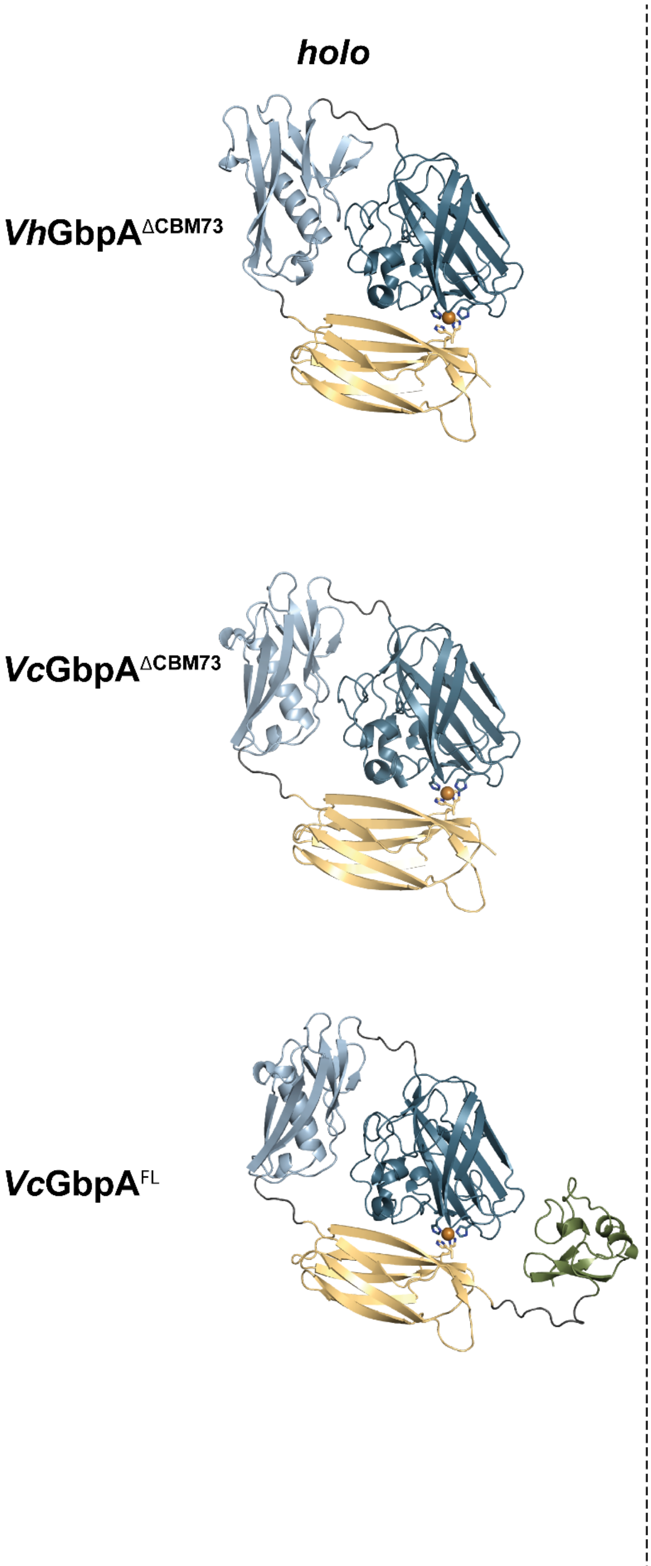

Figure S7. Cartoon representations of *apo*- and *holo*-GbpA structures predicted using AlphaFold3. The predicted copper-loaded structures of *Vh*GbpA$^{\Delta CBM73}$, *Vc*GbpA$^{\Delta CBM73}$, and *Vc*GbpA^{FL} are shown on the left, all showing an interaction between the GbpA3 domain and the catalytic site of the AA10 domain. This interaction occurs independently of the presence of the CBM73 domain. In contrast, the predicted *apo* structures (lacking the copper cofactor) adopt more elongated conformations, and no interaction between the GbpA3 and AA10 domains is predicted.

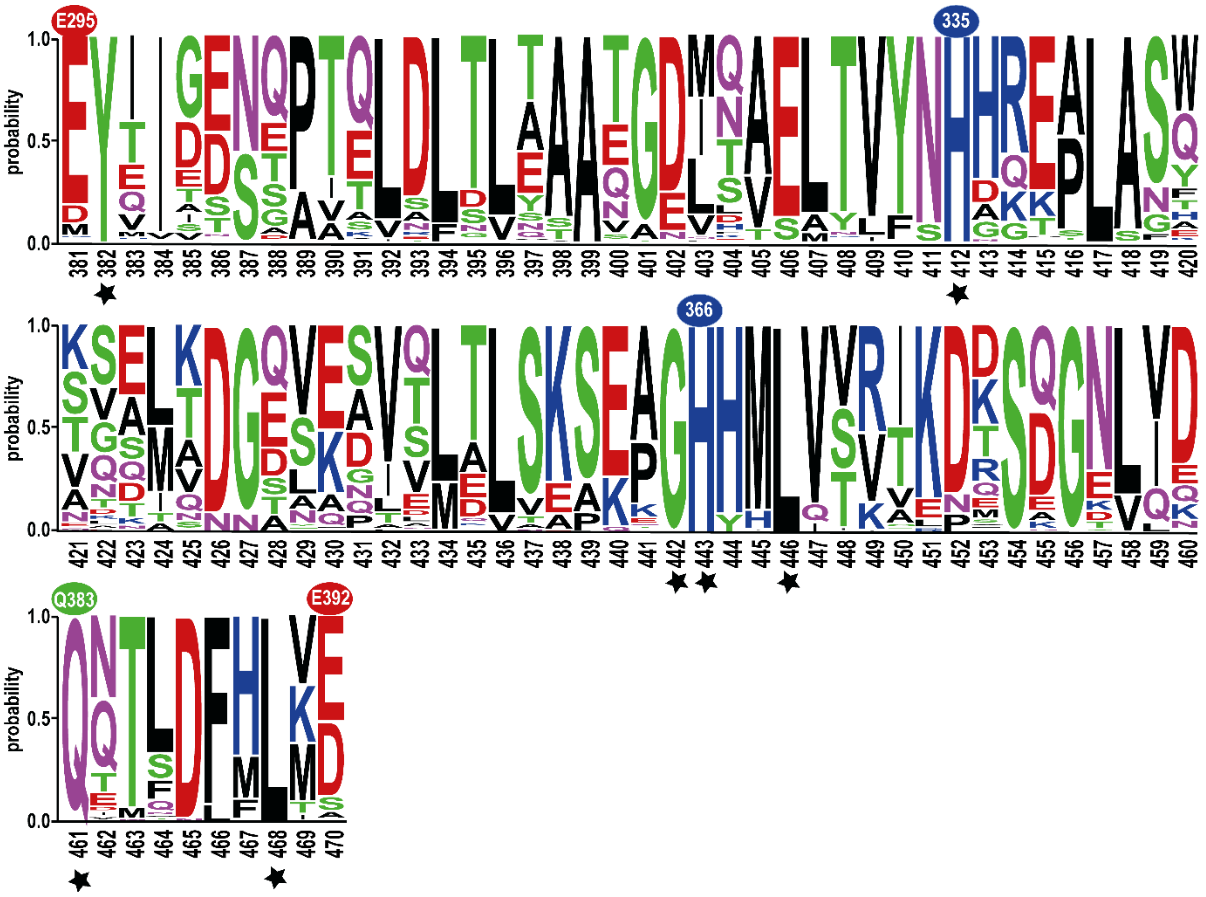

Figure S8. **Amino acid frequency per position based on a multiple sequence alignment of 165 sequences encoding GbpA3 domains associated with LPMOs.** The x-axis shows the MSA position numbering in black. White numbers on red or blue backgrounds highlight specific residues in GbpA3 (numbered according to *Vh*GbpA3): Glu295 and Glu392 mark the N- and C-terminal boundaries of the domain, while His335 and His366 are conserved histidines predicted to coordinate the active site copper in the AlphaFold3 model. A green background marks Gln383, which is discussed in the main text as a potential interaction point with Arg20 and Glu39 in the catalytic domain. Note that residue numbering in *Vh*GbpA excludes the signal peptide; thus, the first histidine in the mature protein is referred to as His1. Black stars beneath the x-axis indicate positions that are 100% conserved across all 165 sequences. The graph was generated using WebLogo [1].

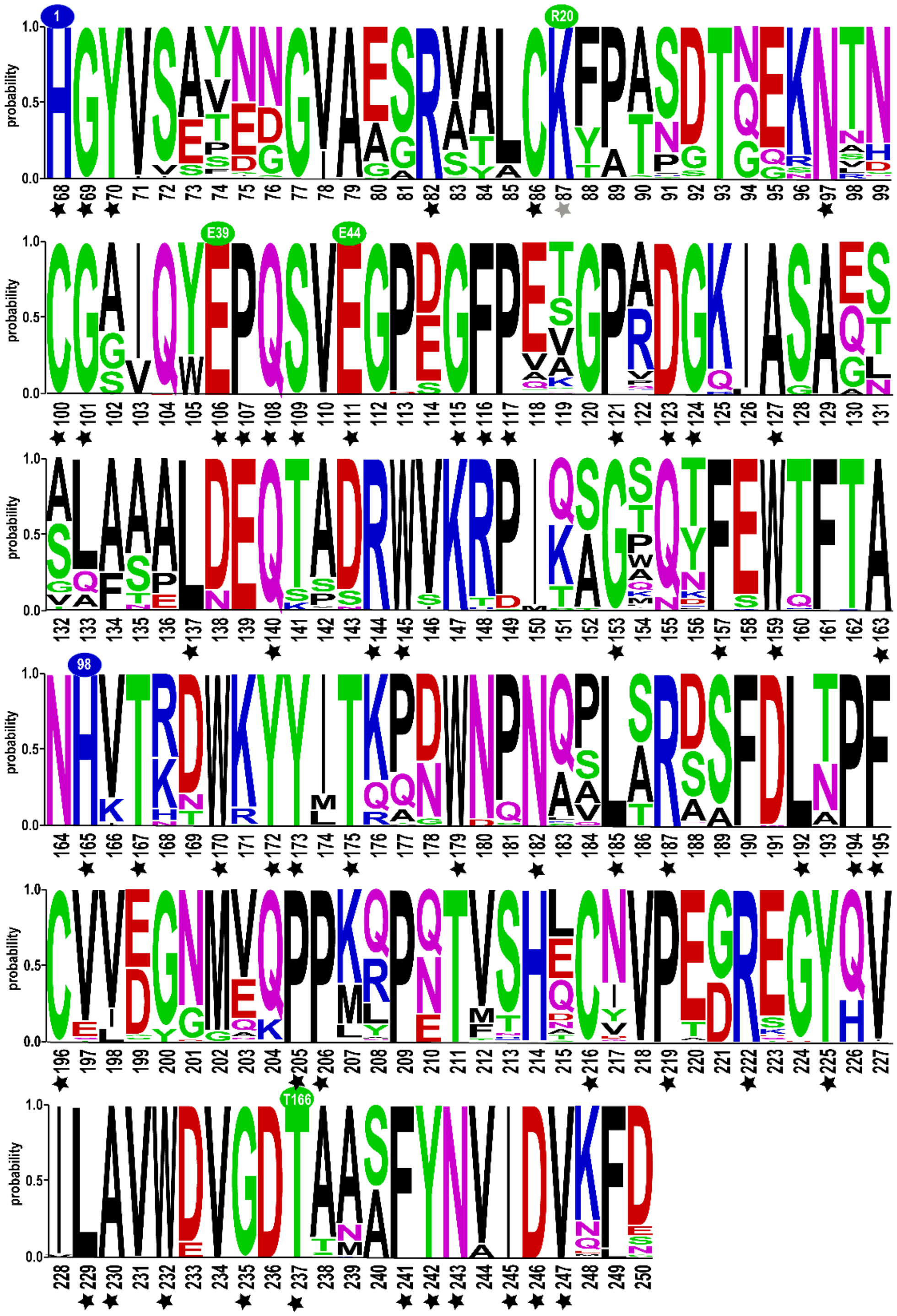

Figure S9. Amino acid frequency per position in the AA10 domains of 165 GbpA-like sequences. The x-axis shows the MSA position numbering in black. White numbers on blue backgrounds highlight the copper-binding histidines, His1 and His98, while white numbers on a green background indicate residues potentially involved in interactions between the AA10 and GbpA3 domains, as discussed in the main text and illustrated in **Fig. S10**. Black stars beneath the x-axis indicate positions that are fully conserved (100%) across all 165 sequences. The grey star below R20 highlights an exception found in *Vh*GbpA, where this position is occupied by an arginine instead of the lysine present in the remaining 164 sequences. Despite this substitution, the side-chain properties and positive charge are retained. The overall pairwise sequence identity across the full-length proteins (comprising all four domains) ranges

from 45.4% to 99.2%, with an average pairwise identity of $51.0 \pm 11.7\%$. The graph was generated using WebLogo [1].

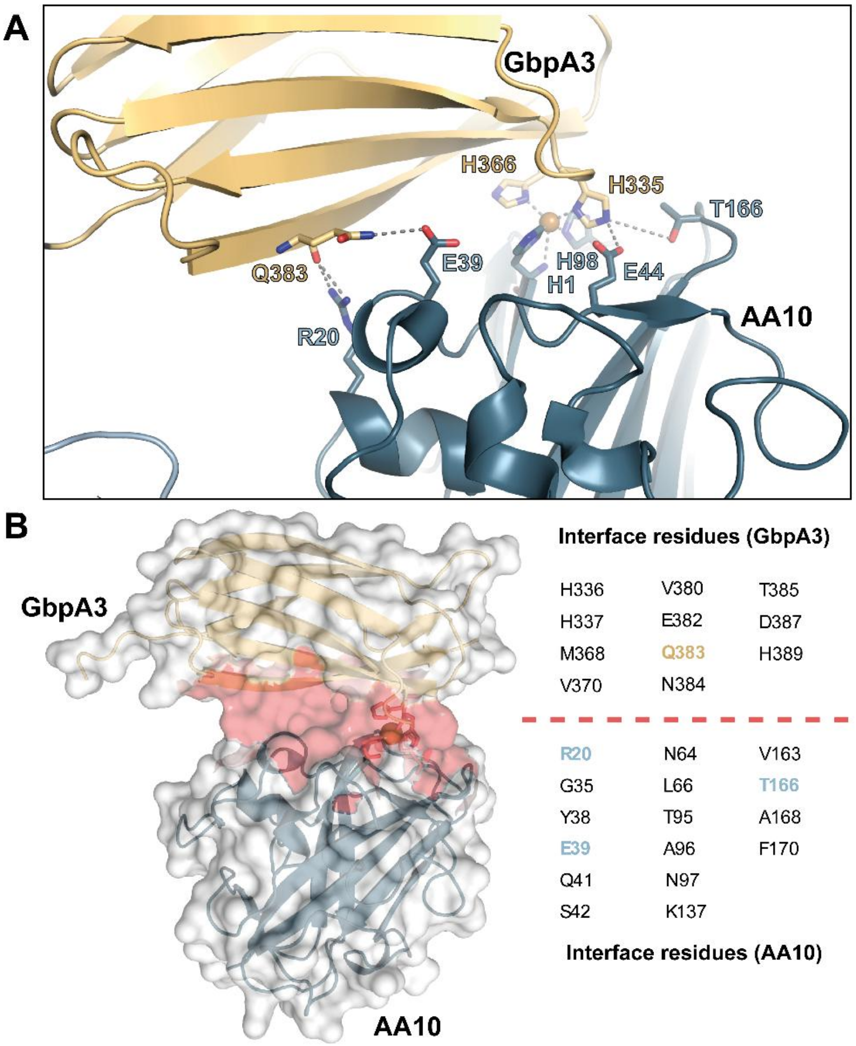

Figure S10. Possible interactions between the GbpA3 domain and the AA10 domain in *holo*-*Vh*GbpA. Panel A shows the AlphaFold-predicted structure of *Vh*GbpA^{FL}, with the AA10 domain colored blue and the GbpA3 domain colored in light sand. Highlighted residues represent potential additional contact points beyond the two copper-coordinating histidines (His335 and His366). These residues are conserved among the 165 GbpA-like sequences used to generate the WebLogos shown in **Figs. S8** and **S9**. Panel B shows the interface area for the AA10-GbpA3 interaction in *Vh*GbpA, colored in red, determined using the PDBePISA server [2, 3]. The right half of panel B lists residues involved in this interaction, beyond the copper-interacting ones (His1 and His98 on the AA10, and His335 and His366 on GbpA3). Residues colored light sand (in GbpA3) or blue (in the LPMO) stood out as important during manual inspection of the complex and are shown in panel A and discussed in the main text. Somewhat surprisingly, the PISA analysis did not pick up on Glu44 (shown in panel A) being important.

3. Supplementary Table

Table S1. The oxidase rate of LPMO variants. Reaction mixtures containing 0.1mM Amplex Red, 5 U.mL^{-1} HRP, and 1 µM enzyme in 20 mM Tris-HCl buffer (pH 7.5) were pre-incubated at 30°C for 5 mins. The reaction was initiated by adding 1mM AscA (final concentration). The solutions were mixed by shaking the plate at 600 rpm for 30 s, and absorption at 563 nm was measured every 30 s for 60 mins. A control reaction lacking the enzyme was included. Oxidase rates were derived from linear progress curves and were corrected for the signal obtained in the control reaction lacking the LPMO. The values represent the mean ± standard deviation for three independent replicates.

	**H_2O_2 production rates (nM.s^{-1})**		
	Full-length	AA10 CD	ΔCBM73
*Vh*GbpA	3.4 ± 0.1	11.0 ± 0.2	5.9 ± 0.1
*Vc*GbpA	3.0 ± 0.1	8.8 ± 0.2	2.5 ± 0.1

4. Supplementary References

(1) Crooks, G. E.; Hon, G.; Chandonia, J. M.; Brenner, S. E. WebLogo: a sequence logo generator. *Genome Res* **2004**, *14* (6), 1188-1190. DOI: 10.1101/gr.849004.

(2) Krissinel, E.; Henrick, K. Inference of macromolecular assemblies from crystalline state. *J Mol Biol* **2007**, *372* (3), 774-797. DOI: 10.1016/j.jmb.2007.05.022.

(3) Jones, S.; Thornton, J. M. Principles of protein-protein interactions. *Proc Natl Acad Sci U S A* **1996**, *93* (1), 13-20. DOI: 10.1073/pnas.93.1.13.
